# Functional characterization and regulatory mechanism of wheat CPK34 kinase in response to drought stress

**DOI:** 10.1186/s12864-020-06985-1

**Published:** 2020-08-24

**Authors:** Ge-Zi Li, Han-Xiao Li, Meng-Jun Xu, Peng-Fei Wang, Xiang-Hong Xiao, Guo-Zhang Kang

**Affiliations:** 1 National Engineering Research Centre for Wheat, Henan Agricultural University, #15 Longzihu College District, Zhengzhou, 450046 Henan Province People’s Republic of China; 2 National Key Laboratory of Wheat and Maize Crop Science, Henan Agricultural University, #15 Longzihu College District, Zhengzhou, 450046 Henan Province People’s Republic of China

**Keywords:** Calcium-dependent protein kinase, Proteomics, BSMV-VIGS, *Triticum aestivum* L., Drought

## Abstract

**Background:**

Drought is one of the most adverse environmental factors limiting crop productions and it is important to identify key genetic determinants for food safety. Calcium-dependent protein kinases (CPKs) are known to be involved in plant growth, development, and environmental stresses. However, biological functions and regulatory mechanisms of many plant CPKs have not been explored. In our previous study, abundance of the wheat CPK34 (TaCPK34) protein was remarkably upregulated in wheat plants suffering from drought stress, inferring that it could be involved in this stress. Therefore, here we further detected its function and mechanism in response to drought stress.

**Results:**

Transcripts of the *TaCPK34* gene were significantly induced after PEG-stimulated water deficiency (20% PEG6000) or 100 μM abscisic acid (ABA) treatments. The *TaCPK34* gene was transiently silenced in wheat genome by using barley stripe mosaic virus-induced silencing (BSMV-VIGS) method. After 14 days of drought stress, the transiently TaCPK34-silenced wheat seedlings showed more sensitivity compared with control, and the plant biomasses and relative water contents significantly decreased, whereas soluble sugar and MDA contents increased. The iTRAQ-based quantitative proteomics was employed to measure the protein expression profiles in leaves of the transiently TaCPK34-silenced wheat plants after drought stress. There were 6103 proteins identified, of these, 51 proteins exhibited significantly altered abundance, they were involved in diverse function. And sequence analysis on the promoters of genes, which encoded the above identified proteins, indicated that some promoters harbored some ABA-responsive elements. We determined the interactions between TaCPK34 and three identified proteins by using bimolecular fluorescent complementation (BiFC) method and our data indicated that TaCPK34directly interacted with the glutathione S-transferase 1 and prx113, respectively.

**Conclusions:**

Our study suggested that the *TaCPK34* gene played positive roles in wheat response to drought stress through directly or indirectly regulating the expression of ABA-dependent manner genes, which were encoding identified proteins from iTRAQ-based quantitative proteomics. And it could be used as one potential gene to develop crop cultivars with improved drought tolerance.

## Background

Because of the ever-increasing global population and the climate change, the food safety becomes a major issue because it needs an annual increase (approximately 2.4%) in food yields, whereas yields of the major crops wheat, rice, and maize are presently increased merely only at 0.9, 1 and 1.6%, respectively [1]. Abiotic stresses including drought, cold, heat, and salt are major adverse environmental stresses that greatly limit crop yields and quality. Therefore, it is pivotal to understand the molecular mechanisms underlying stress responses,identify important genetic genes and develop crop cultivars with enhanced stress tolerance.

Plants respond to environmental stresses by regulating the expression of numerous stress-responsive genes through complicated signaling pathways. Ca^2+^ has been recognized as a major second messenger in these pathways, and can perceive intracellular changes in Ca^2+^ concentrations and translate them into specific phosphorylation events to initiate downstream signaling processes [2]. In plants, several classes of Ca^2+^ sensors: calcium-dependent protein kinases (CPKs or CDPKs), calmodulins (CaMs) and CaM-like proteins and calcineurin B-like proteins (CBLs) have been reported to involve in the perception and transmission of intracellular Ca^2+^ concentrations [3]. CaMs and CBLs do not have enzymatic activities and therefore do not directly transmit the Ca^2+^ signals, whereas CPKs can sense Ca^2+^ signals and directly mediate a variety of cellular responses [4].

CPKs, a large multigene family of Ca^2+^-regulated Ser/Thr protein kinases, contain four-domain structure consisting of a variable N-terminal variable domain, a kinase or catalytic domain, a CPK activation domain, and a CaM-like domain that is composed of four EF hand motifs [5]. CPKs are involved in biotic and abiotic signal transduction pathways through recognizing the Ca^2+^ signatures [2, 6]. The numbers within this kinase family vary among different plant species [7]. For instance, 34, 31, 30, and 20 CPKs have been identified members in *Arabidopsis thaliana*, rice, *Brachypodium distachyon*, and bread wheat, respectively [8–10]. Different CPK members could have differential roles in response to environmental stresses and even within the same stress. For example, *AtCPK6* and *AtCPK10* in *Arabidopsis* and *OsCPK13* in rice have been found to positively regulate the drought tolerance, respectively [11–13], whereas *AtCPK23* negatively regulates drought and salt tolerance [14].

Growing evidence has demonstrated the crucial functions of CPKs in plant growth, development, and responses to environmental stresses, whereas the biological functions and regulatory mechanisms of many plant CPKs remain unclear [9, 15]. Bread wheat (*Triticum aestivum* L., hexaploid species, AABBDD) is one of the most important global cereal crops and offers a rich source of carbohydrates, proteins, minerals, and other nutrients essential for the human diet [16]. Our previous study identified genome-scale protein expression profiles of wheat grains after anthesis. Among the identified proteins, a wheat CPK protein (CPK34) sharply increased as grain development proceeded, inferring that it could be involved in grain filling and environmental stress tolerance [17]. To our knowledge, however, the function and mechanism of TaCPK34 in response to environmental stresses has not been examined [9, 15]. The objectives of this study were (1) to determine the role of TaCPK34 in wheat response to drought stress by using the BSMV-VIGS method and (2) to investigate the potential molecular mechanisms by which TaCPK34 influences drought tolerance by using the isobaric tagging for relative and absolute quantification (iTRAQ) proteomic analysis.

## Results

### Identification of TaCPK34

During the wheat grain filling period (from 22 April to 31 May) in 2015, our lab performed a quantitative proteomics to characterize the genome-scale grain protein expression profiles of the bread wheat cultivar Zhoumai 18 at different grain filling stages (5, 10, 15, 20, 25, 30, and 35 days after anthesis) under field conditions [17]. During the late stages of wheat grain filling period (from 6 to 31 May, 2015, corresponding to 15 ~ 35 days after anthesis), there was only 25.5 mm rainfall, which was enough for wheat growth and caused drought stress in this growth season (Table S1). From these proteomic data, one protein that matched to TuCPK34 (GenBank accession no. EMS58754) were identified from *Triticum urartu* (diploid, AA, the progenitor of the A subgenome of hexaploid bread wheat), and its sequence of amino acid alignment showed 87% similarity to Chinese Spring (CS) (Figure S1), suggesting that TaCPK34 was the homologue of TuCPK34 in *Triticum aestivum* (Figure S1). Compared to the protein abundance of TaCPK34 at 5 days after anthesis (control), its abundance in wheat grains at the subsequent sampling timepoints (15, 20, 30, and 35 days after anthesis) was more than 16-fold higher (Figure S2).

### Characterization and subcellular localization of TaCPK34

**Fig. 1:**
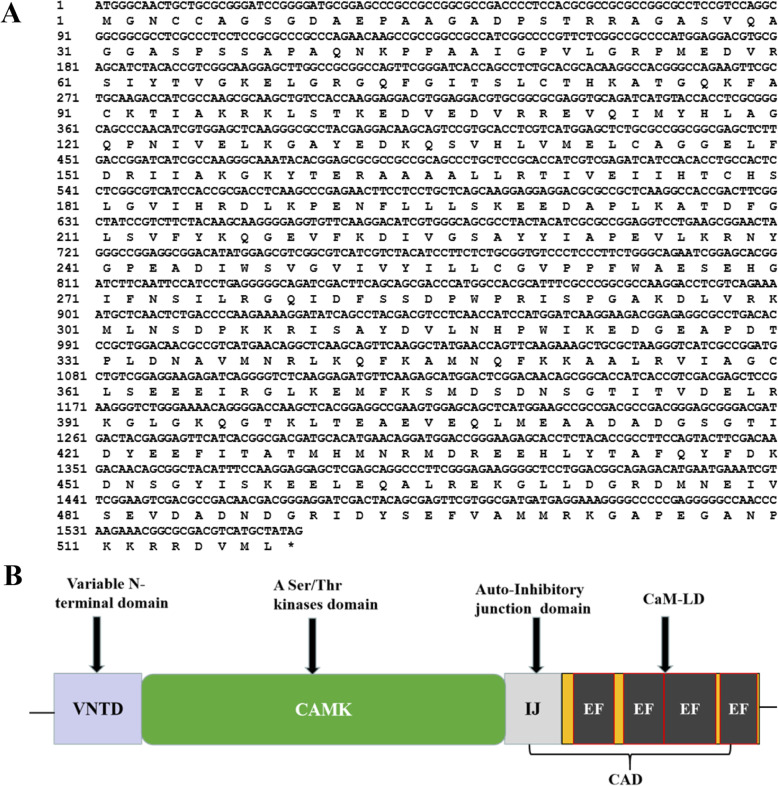
Sequences and domain structure of TaCPK 34 protein. **a**, Nucleotide and amino acid sequences of TaCPK34. **b**, Domain structure of TaCPK 34 protein. Plant CPK members consist of variable N terminal domain (VNTD) containing N-myristoylation/N-palmitoylation region fused to a catalytic kinase domain, an inhibitory junction domain and a calmodulin like domain (CaM-LD). Inhibitory junction and CaM-LD forms CPK activation domain (CAD). CaMLD contains four calcium binding EF hands motifs that are organized in N terminal and C terminal lobes.section may be divided by subheadings. It provides a concise description of the experimental results, their interpretation as well as the experimental conclusions are drawn

**Fig. 2:**
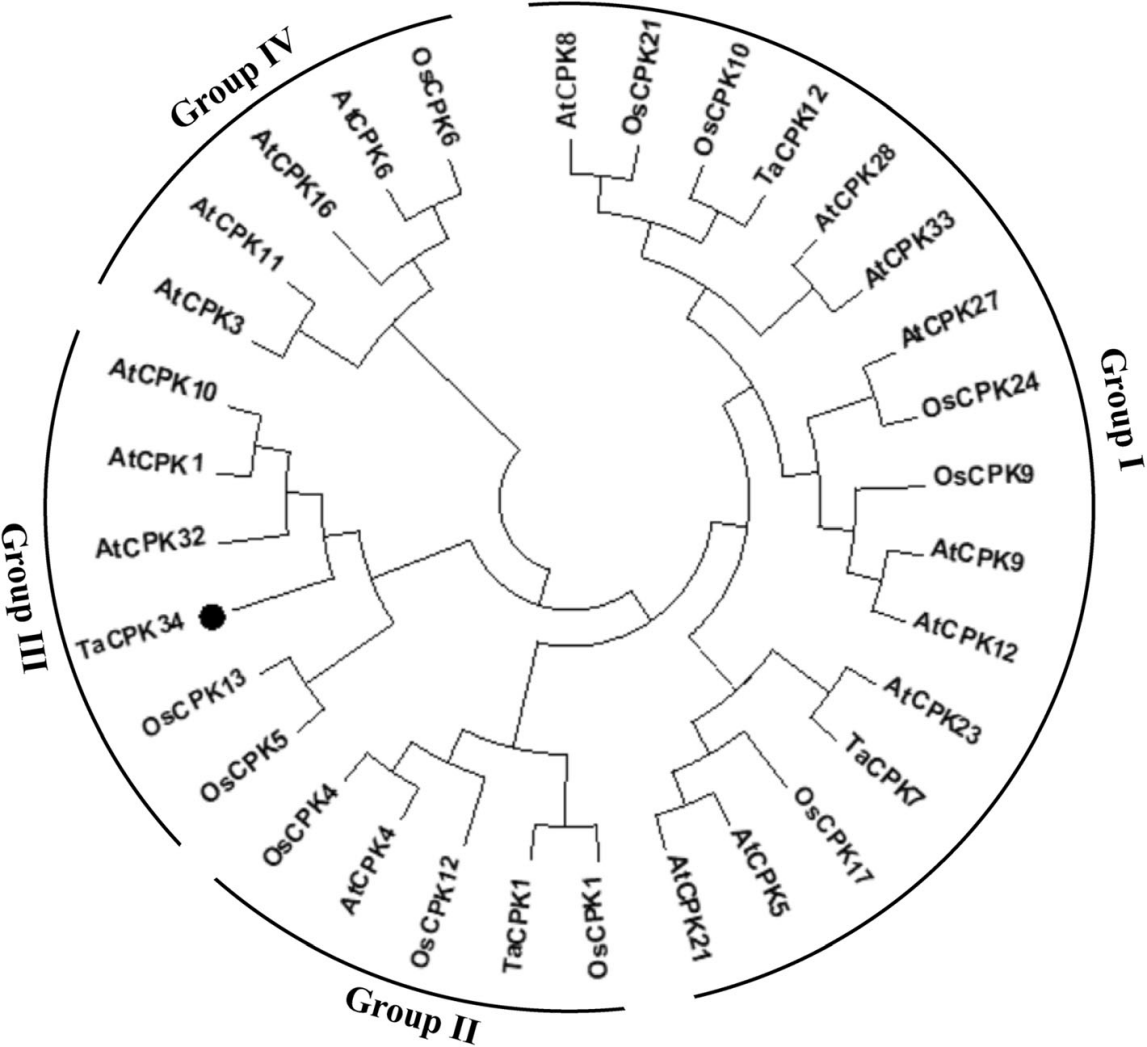
Phylogenetic tree of TaCPK34 protein. Function-known CPKs in bread wheat and two model plants (*Arabidopsis thaliana* and rice) are used to construct their phylogenetic tree by using MEGA7.0 [9]. Optimal tree with the sum of branch length = 35.81 is shown. TaCPK34 is indicated by black circles

The cDNA sequence of *TaCPK34* was isolated and sequenced (Fig. 1a), its predicated protein had 518 amino acids (Fig. 1a), and contained the three typical domains of plant CPKs: a variable N-terminal domain, a serine/threonine kinase domain, and a CPK activation domain that comprised an inhibitory junction domain and a C-terminal Ca^2+^-binding domain (Fig. 1b). Phylogenetic tree of TaCPK34 protein was constructed and grouped into four branches (groups I-IV) (Fig. 2), which followed CPKs’ phylogenetic tree in rice and *Arabidopsis thaliana* [9, 15], using CPKs of the identified wheat (3 CPKs), rice (11 CPKs) and *Arabidopsis thaliana* (17 CPKs) (Table S2). The TaCPK34 protein shared high similarities (≥95%) with AtCPK32, AtCPK1, and AtCPK10 of group III (Fig. 2).

### Expression patterns of *TaCPK34* gene in responses to water deficiency and ABA

**Fig. 3:**
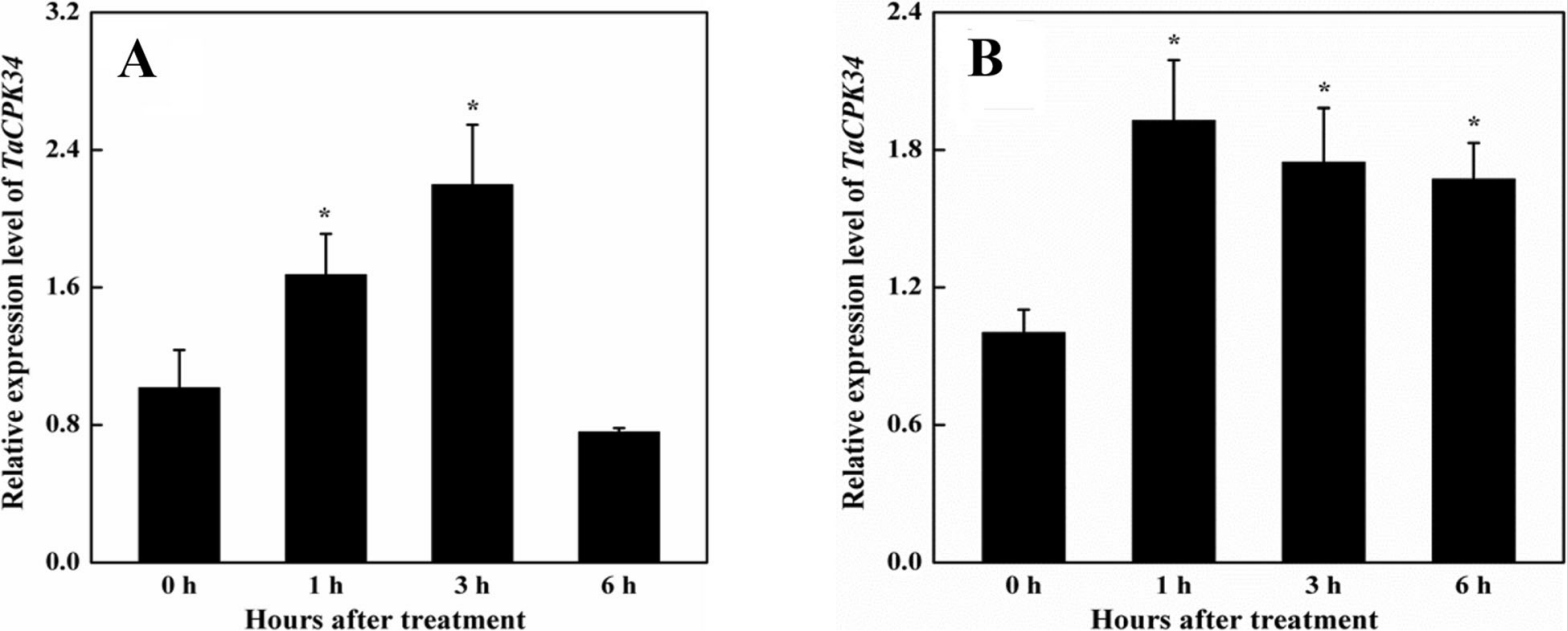
Transcripts of the *TaCPK34* gene in leaves. Wheat plants were suffering from 20% PEG6000-stimulated water deficiency (**a**) and 100 μM ABA (**b**). Transcripts are determined by using qPCR and the *TaActin* gene is used as the internal control. Each value is the mean ± standard deviation of at least three independent measurements. Asterisks indicate statistically significant differences (*P* < 0.05)

PEG-stimulated water deficiency (20% PEG6000) and 100 μM abscisic acid (ABA) were applied to wheat seedlings to monitor the transcriptional responses of the *TaCPK34* gene. Under water deficiency condition, transcripts of the *TaCPK34* gene increased quickly, reached the highest level at 3 h, and then decreased quickly to low levels at 6 h (Fig. 3a). After a 100 μM ABA treatment, the transcripts of the *TaCPK34* gene quickly increased, remarkably higher than that in control seedlings (0 h) at all sampling timepoints (1, 3, and 6 h) (Fig. 3b).

### Role of TaCPK34 in response to water deficiency

Barley stripe mosaic virus-induced silencing (BSMV-VIGS) method was used to verify the function of TaCPK34 under drought stress. A 219 bp-length conserved cDNA fragment (+ 505 to + 723, the translation start site “ATG” is + 1) of *TaCPK34* gene, which shared high identity (100%) with the other two copies (Figure S3), was used to construct its silencing vector (BSMV-VIGS-TaCPK34), and BSMV-VIGS-GFP vector was used as the control. The silencing vectors of BSMV-VIGS-TaCPK34 and BSMV-VIGS-GFP were separately inoculated into the leaves of wheat seedlings. At 6 days after inoculation, BSMV-VIGS-induced chlorosis emerged on the leaves of both BSMV-VIGS-TaCPK34- or BSMV-VIGS-GFP-inoculated wheat seedlings (Figure S4A). Transcripts of the *TaCPK34* gene were remarkably inhibited in leaves of BSMV-VIGS-TaCPK34-inoculated wheat seedlings with chlorotic symptoms (Figure S4B).

**Fig. 4:**
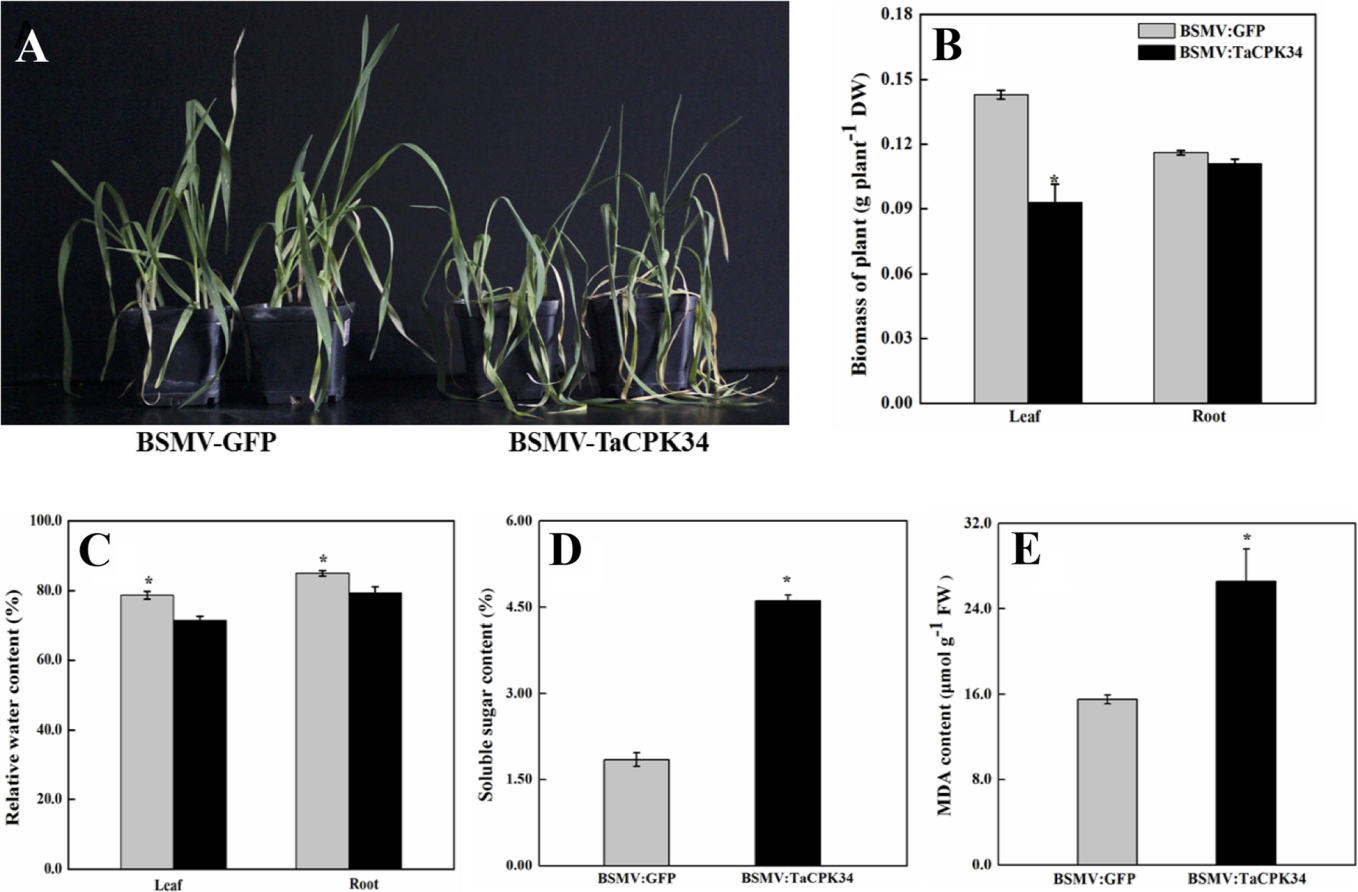
Function of the *TaCPK34* gene in response to drought stress. **a**, Phenotypes of BSMV-VIGS-TaCPK34- or BSMV-VIGS-GFP-inoculated wheat seedlings are observed at 14 days after drought stress. Biomass of dry weight (**b**), relative water content (**c**), soluble sugar content (**d**), and MDA content (**e**) of leaves in BSMV-VIGS-TaCPK34-inoculated wheat seedlings and BSMV-VIGS-GFP-inoculated wheat seedlings are determined at 14 days after drought stress. Each value is the mean ± standard deviation of at least three independent measurements. Asterisks indicate statistically significant differences (*P* < 0.05)

At 6 days after inoculation, inoculated wheat seedlings with chlorotic symptoms were well watered, and then suffered from water loss stress (drought) for subsequent 14 days. At this timepoint, the BSMV-VIGS-TaCPK34-inoculated wheat plants exhibited visual drought-induced damage symptoms such as leaf rolling and wilting, whereas the BSMV-VIGS-GFP-inoculated wheat seedlings remained vigorous (Fig. 4a). Coincident with these qualitative phenotypic changes, plant biomasses and relative water contents in BSMV-VIGS-TaCPK34-inoculated wheat plants were significantly lower than those in BSMV-VIGS-GFP-inoculated wheat seedlings (Fig. 4b and c). However, the contents of soluble sugar and MDA were remarkably higher in the former than those in the latter (Fig. 4d and e).

### Molecular mechanism of TaCPK34-mediated drought tolerance

**Fig. 5:**
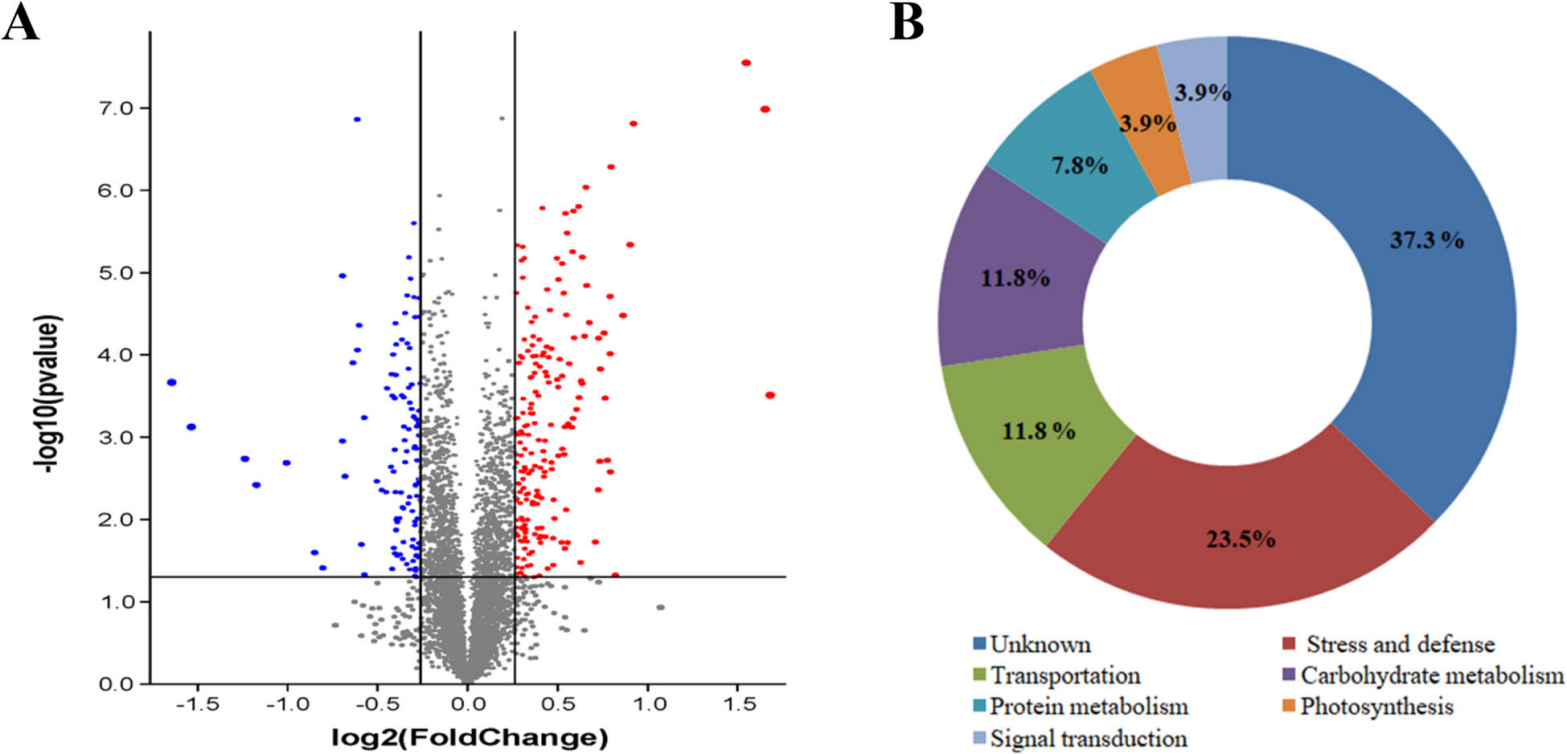
Volcano plot (**a**) and functional categorization (**b**) of the identified proteins based on iTRAQ proteomics. The iTRAQ proteomics is performed on leaves of BSMV-VIGS-TaCPK34- or BSMV-VIGS-GFP-inoculated wheat plants suffering drought stress for 14 days. And the different colors of circles represent that there were significant difference between samples, the blue circle represents down-regulated proteins, red circle represents up-regulated proteins, grey circle represents none-regulated (not significant difference of samples) proteins, respectively

The iTRAQ-based quantitative proteomics was employed to identify differences in the leaf protein profiles between the BSMV-VIGS-TaCPK34- and the BSMV-VIGS-GFP-inoculated wheat seedlings at 14 days after drought stress. A total of 25,017 peptides were identified from 295,102 mass spectra (Table S3). And these corresponded to 6, 103 expressed proteins, which were detected in all four biological replicates in the above treatments (Table S4). Our mass spectrometry proteomics data have been deposited (ProteomeXchange identifier PXD008567). Ratios were used to assess the fold changes in the abundance of proteins between the BSMV-VIGS-TaCPK34- and BSMV-VIGS-GFP-inoculated wheat plants. A volcano plot illustrated the asymmetry between up-regulated (red) and down-regulated (blue) differentially expressed proteins, and the correlation of labeled samples showed good reproducibility between the four biological replicates (Fig. 5a). Fold changes of ≥1.50- or ≤ 0.67-fold were considered to be significant in the abundance of identified proteins, and 51 proteins with significantly altered abundance (*P* < 0.05 with 95% confidence, *FDR* < 0.05) were identified between BSMV-VIGS-TaCPK34- and BSMV-VIGS-GFP-inoculated wheat seedlings suffering from the above drought stress (Table 1).

**Table 1:** Identification of the differentially expressed proteins in leaves of BSMV-VIGS-TaCPK34-inoculated wheat plants

Accession no. (NCBI)	Protein names	Average ratio	T-test	FDR (BH test)
**Stress and defense**				
XP_020155660.1	Dehydrin DHN2	3.21 ± 0.59	3.07E-04	9.97E-03
ALD18912.1	LEA3 protein	2.92 ± 0.10	2.83E-08	1.73E-04
XP_020162307.1	Low-temperature-induced 65 kDa protein-like	1.70 ± 0.14	3.34E-04	1.03E-02
XP_020147546.1	Barwin-like	1.73 ± 0.14	9.70E-05	5.76E-03
XP_020190115.1	11 kDa late embryogenesis abundant protein-like	1.66 ± 0.11	1.96E-03	2.81E-02
EMT24839.1	Dehydrin DHN 3	1.60 ± 0.11	4.05E-05	3.51E-03
EMS55485.1	Germin-like protein 8-4	1.58 ± 0.04	9.21E-07	8.04E-04
ALD18913.1	LEA 3	1.58 ± 0.07	1.43E-05	2.43E-03
XP_020167477.1	Germin-like protein 4-1	0.66 ± 0.03	4.38E-05	3.63E-03
ACF08092.1	Prx113	0.65 ± 0.01	8.75E-05	5.46E-03
P30110.1	Glutathione S-transferase 1	0.62 ± 0.06	1.11E-03	2.04E-02
CAA39486.1	Peroxidase	0.62 ± 0.03	1.09E-05	2.10E-03
**Signal transduction**				
XP_020175672.1	Serine carboxypeptidase 2-like	3.21 ± 0.78	2.99E-03	8.72E-01
EMS46272.1	Serine/threonine-protein kinase CTR1	1.73 ± 0.29	2.64E-03	3.29E-02
**Carbohydrate metabolism**				
XP_020159606.1	Transketolase	3.21 ± 0.77	5.77E-04	1.49E-02
XP_020174663.1	UDP-glycosyltransferase 88B1-like	3.21 ± 0.79	3.86E-02	1.63E-01
XP_020189214.1	Probable cinnamyl alcohol dehydrogenase 5	3.21 ± 0.83	1.37E-07	1.88E-04
XP_020199323.1	Putative acyl-activating enzyme 19 isoform X1	3.21 ± 0.80	2.51E-02	1.30E-01
XP_020152513.1	Probable xyloglucan endotransglucosylase	3.14 ± 0.12	1.03E-07	1.88E-04
XP_020201268.1	Short-chain dehydrogenase TIC 32	1.63 ± 0.37	1.87E-02	2.44E-01
**Photosynthesis**				
EMT23001.1	Putative chlorophyll a-b binding protein 1C	1.55 ± 0.03	2.21E-04	8.60E-03
XP_020164958.1	Outer envelope pore protein 16-2	1.52 ± 0.09	4.57E-04	1.28E-02
**Protein metabolism**				
XP_020185387.1	Bisdemethoxycurcumin synthase-like	1.69 ± 0.06	5.41E-05	4.29E-03
XP_020158799.1	Berberine bridge enzyme-like 27	1.55 ± 0.12	2.07E-04	8.32E-03
XP_020167595.1	Aspartyl protease AED3-like	1.50 ± 0.06	5.54E-06	1.62E-03
EMT21970.1	Insulin-degrading enzyme	0.50 ± 0.17	2.05E-03	2.84E-02
**Transportation**				
XP_020175501.1	Protein TRIGALACTOSYLDIACYLGLYCEROL 3	3.21 ± 0.81	7.49E-04	4.75E-01
XP_020197700.1	Non-specific lipid-transfer protein 4.1-like	1.87 ± 0.04	4.55E-06	1.56E-03
XP_020167906.1	Non-specific lipid-transfer protein 2P-like	1.82 ± 0.11	3.31E-05	3.21E-03
XP_020151640.1	Non-specific lipid-transfer protein 3-like isoform X1	1.74 ± 0.05	5.19E-07	5.29E-04
CAH69190.1	Type 1 non specific lipid transfer protein precursor	1.67 ± 0.14	1.49E-04	7.12E-03
CAH69206.1	Type 1 non specific lipid transfer protein precursor	1.55 ± 0.07	6.46E-06	1.62E-03
**Unknown**				
EMT11521.1	Hypothetical protein F775_17917	1.89 ± 0.04	1.54E-07	1.88E-04
CDM80815.1	Unnamed protein product	1.77 ± 0.33	4.74E-02	1.83E-01
EMS62850.1	Hypothetical protein TRIUR3_20788	1.73 ± 0.11	1.94E-05	2.60E-03
EMS51097.1	Hypothetical protein TRIUR3_06539	1.71 ± 0.23	1.91E-03	2.75E-02
2003xXP_020154832.1	Uncharacterized protein LOC109740203	1.66 ± 0.12	6.28E-05	4.57E-03
XP_020160295.1	Uncharacterized protein LOC109745583	1.65 ± 0.28	4.35E-03	4.43E-02
BAJ88864.1	Predicted protein	1.57 ± 0.08	5.93E-05	4.48E-03
XP_020199551.1	Uncharacterized protein LOC109785365	1.54 ± 0.12	3.32E-02	1.51E-01
XP_020173255.1	Uncharacterized protein LOC109758798	1.53 ± 0.04	1.58E-06	8.98E-04
XP_020191925.1	Uncharacterized protein LOC109777773	1.51 ± 0.07	6.22E-05	4.57E-03
XP_020166692.1	Uncharacterized protein LOC109752199	1.50 ± 0.05	1.79E-06	8.98E-04
XP_020198515.1	Uncharacterized protein LOC109784334	1.50 ± 0.13	5.89E-04	1.49E-02
EMS52615.1	Hypothetical protein TRIUR3_12537	0.67 ± 0.21	4.66E-02	1.82E-01
EMS56093.1	Hypothetical protein TRIUR3_23412	0.66 ± 0.10	2.00E-02	1.11E-01
XP_020200627.1	Uncharacterized protein LOC109786464	0.64 ± 0.06	1.25E-04	6.36E-03
YP_008239094.1	Hypothetical chloroplast RF34 (chloroplast)	0.44 ± 0.14	3.81E-03	4.06E-02
XP_020170017.1	Uncharacterized protein LOC109755533	0.42 ± 0.12	1.83E-03	2.67E-02
CDM81748.1	Unnamed protein product	0.32 ± 0.04	2.15E-04	1.17E-01

On the basis of their biological and molecular functions, the differentially expressed proteins were classified into two groups of known (32, 62.7%) and unknown (19, 37.3%) functions. The function-known proteins were further divided into six categories: stress and defense (12), signal transduction (2), carbohydrate metabolism (6), photosynthesis (2), protein metabolism (4), and transportation (6) (Fig. 5b).

The promoters of the genes encoding the above function-known proteins were retrieved from the International Wheat Genome Sequencing Consortium (IWGSC) database (RefSeq 1.0 version) from Ensemblplants (http://plants.ensembl.org/index.html). Their sequences were analyzed by using PlantCARE software, and our analysis indicated that the promoter sequences of these genes encoding 19 identified proteins harbored ABA-responsive elements. These genes encoded the dehydrin DHN2, LEA3 protein, low-temperature-induced 65 kDa protein-like isoform, barwin-like, 11 kDa late embryogenesis abundant protein-like, LEA 3, germin-like protein 4–1, Prx113, glutathione S-transferase 1, peroxidase, probable xyloglucan endotransglucosylase, serine/threonine-protein kinase CTR1, putative acyl-activating enzyme 19 isoform X1, aspartyl protease AED3-like, non-specific lipid-transfer protein 4.1-like, non-specific lipid-transfer protein 3-like isoform X1, type 1 non specific lipid transfer protein precursor, short-chain dehydrogenase TIC 32, and type 1 non specific lipid transfer protein precursor (Figure S5).

**Fig. 6:**
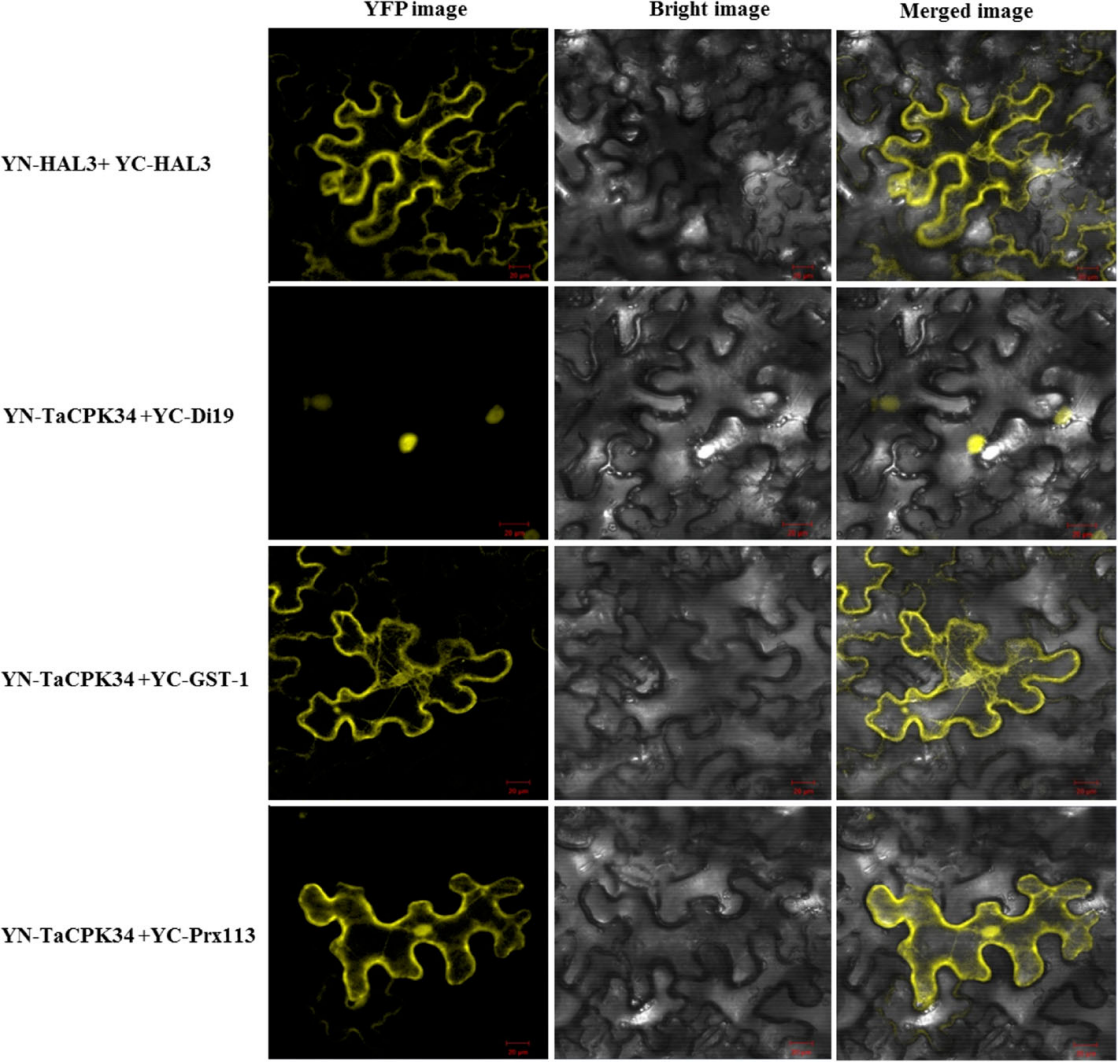
BiFC assay of the interaction between TaCPK34 and TaGST1 or TaPrx113 proteins. YFP fluorescence is detected by confocal laser scanning microscopy. The YN-TaCPK34 and YC-TaGST1 or YC-TaPrx113 constructs are co-infiltrated in tobacco leaves. Co-transformants of HAL3 or Di19 with YFP as well as YN-TaCPK34 and YC are used as positive controls, respectively. Bar = 20 μm

Three stress and defense-related enzymes/proteins (glutathione S-transferase 1, prx113, and peroxidase), which have been reported to directly function in biotic and abiotic stress tolerance [18, 19], were selected for BiFC assay to determine whether they interacted directly with TaCPK34. In this study, TaCPK34 was fused to the N-terminal fragment of YFP, while glutathione S-transferase 1 (GST1), prx113, or peroxidase were fused to the C-terminal fragment of YFP, respectively. If the two tested proteins associated with one another, a complete fluorescent YFP should be detected. Our results showed that YFP fluorescence was observed at the plasma membrane or in nucleus when TaCPK34 was injected together with YC-GST1 or YC-prx113 (Fig. 6), whereas not with YC-peroxidase (data not indicated). These results indicated that TaCPK34 could directly interact with GST1 in the plasma membrane, and prx113 at the plasma membrane and in the nucleus.

## Discussion

### TaCPK34 could be a novel and drought-tolerant plant CPK protein

Evolutionary analyses have revealed that gene duplication (e.g., tandem duplication, segmental duplication) has played an important role in the expansion and evolution of plant CPKs [20–22]. Thus, plant CPKs constitute a multigene family, and there are differences in the numbers and functions of CPK family members in different plant species [9, 15]. Only 20 CPK encoding genes (TaCPK1 ~ 20) have been identified in the very large and complex genome of bread wheat [9, 23]. In the post-genomic era, recently available genomic sequencing data for bread wheat and its relatives [24–28], have helped to explore more members of crucial CPKs. In our previous proteomic data, one peptide was matched to TuCPK34 [17], and its orthologous *TaCPK34* gene was isolated in this study (Fig. 1a). TaCPK34 protein shared low identities (≤ 50%) to other wheat CPKs and belonged to different groups (Fig. 2), suggesting that TaCPK34 might be a novel member of wheat CPKs.

To cope with environmental stress, plants have evolved sophisticated regulatory mechanisms and undergone a series of morphological, physiological, developmental, and cellular changes. At the genetic level, these changes are well controlled byenvironment-tolerant genes, and therefore, the identification of environment-tolerant genes is the first step toward developing the environment-tolerant cultivars [29]. Of the reported 20 wheat CPKs, only 4 (CPK1, 6, 9 and 18) have been found to be drought-responsive, but their drought tolerance has not been tested [30]. In our previous study, the abundance of TaCPK34 protein was upregulated during the late stages of wheat grain filling period (from 6 to 31 May, 2015, corresponding to 15 ~ 35 days after anthesis) under natural field condition (There was only 25.5 mm rainfall, from 6 to 31 May, 2015, thus drought stress occurred), in which wheat plants suffered from various stresses including drought [17]. Transcripts of the *TaCPK34* gene were significantly induced by the PEG-stimulated drought stress (Fig. 3a). These changes in the expression patterns at the translational and transcriptional levels implied the requirement of TaCPK34 in wheat plant for drought adaptation. BSMV-VIGS-TaCPK34- and BSMV-VIGS-GFP-inoculated wheat plants displayed a drought sensitive phenotype with inhibited growth and physiological parameters (Fig. 4). These results suggested that TaCPK34 is a wheat drought-tolerant gene, and could be used for one gene resource for breeding wheat cultivars with drought tolerance.

### TaCPK34 could regulate expression of the target genes in response to drought stress

Stress stimuli, as well as growth and development, induce a series of cellular events, firstly changes in Ca^2+^ concentration to generate specific Ca^2+^ signatures and subsequently activate Ca^2+^ sensors, then activated Ca^2+^ sensors, which decoded the Ca^2+^ signatures and induced downstream reactions, including changes in gene expression [6, 15]. However, there are functional specificities among multiple Ca^2+^ sensors due to their differential structures, which allow them to sense specific Ca^2+^ signatures or targeting specific and unique genes/proteins [9, 30]. CPKs are major Ca^2+^ sensors in plant cells, and play important roles in plants adaptation to stress stimuli by detecting the stress signals and inducing the expression of downstream genes. From the view of genetic engineering, therefore, they could be crucial for conferring stress tolerance in crop plants. To our knowledge, however, only few plant CPKs have functionally been confirmed to mediate drought tolerance in plants and in two model plants, these include *Arabidopsis thaliana* CPK4, 6, 8, 10, 11, and 21 [4, 11, 13, 31, 32], and *Oryza sativa* CPK1, 4, 9, and 10 [1, 33–35]. Of these CPKs, TaCPK34 had high similarities to AtCPK10, and they were grouped in the same group (III) (Fig. 2), suggesting that they could have similar mechanisms. AtCPK10 has been found to interact with HSP1 and participate the drought tolerance through the modulation of ABA- and Ca^2+^-mediated stomatal movements [10]. OsCPK4 enhances the drought tolerance through reducing membrane lipid peroxidation in rice, possibly by upregulating a number of genes associated with the lipid metabolism [36]. However, molecular mechanisms underlying their function in drought tolerance are largely unknown. Here, potential molecular mechanisms of TaCPK34-mediated drought tolerance in wheat were explored by using the iTRAQ-based proteomic approach.

Our proteomic experiments identified 51 proteins with significantly altered abundance (Fold changes of ≥1.50- or ≤ 0.67, *P* < 0.05) in BSMV-VIGS-TaCPK34-inoculated wheat plants (Table 1). These identified proteins were grouped into two types of known and unknown functions. A large proportion (19/51, 37.3%) of the unknown function proteins were not annotated (Fig. 5b, Table 1), possibly because the genome of bread wheat is huge and complex, and many of its genes/proteins have not been characterized. This has slowed research progress in wheat compared with its homologous species such as rice and maize, whose genomes are relatively small and simple [37]. The identified proteins with known function were involved with stress defense, signal transduction, protein metabolism, carbohydrate metabolism, etc. (Table 1). It has been reported that several plant CPKs function in drought stress responses through direct interaction with some proteins. In *Arabidopsis*, CPK8 has been found to serve as a positive regulator in plant responses to drought stress through the direct interaction with CAT3 protein to regulate its activity [4]. More evidences have indicated that many CPKs indirectly control the expression of a series of stress-responsive genes through various regulators [9, 14]. OsCPK14 has been identified to interact with OsDi19–4, one member of the drought-induced 19 (Di19) transcription factor family, whose overexpressing *Arabidopsis* plants display enhanced drought tolerance. Di19 bound to the promoters of *OsASPG1* and *OsNAC18* genes, and thus, OsCPK14 indirectly regulated the expression of these two genes through Di19 [38]. Among our identified proteins of known function, many proteins or their isoforms have been reported to function in drought stress and they included dehydrin [39], LEAs [40], peroxidases [18], GST1 [19], germin-like proteins [41], low-temperature-induced 65 kDa protein [42]. These implied that TaCPK34 could directly or indirectly regulate the expression of the genes encoding these proteins. Our BiFC assay showed that TaCPK34 directly interacted with GST1 and prx113 (Fig. 6), while not with peroxidase, suggesting that it could directly regulate the expression of the formers, while indirectly control the latter. The direct or indirect relations between TaCPK34 and other identified proteins need to be determined.

**Fig. 7:**
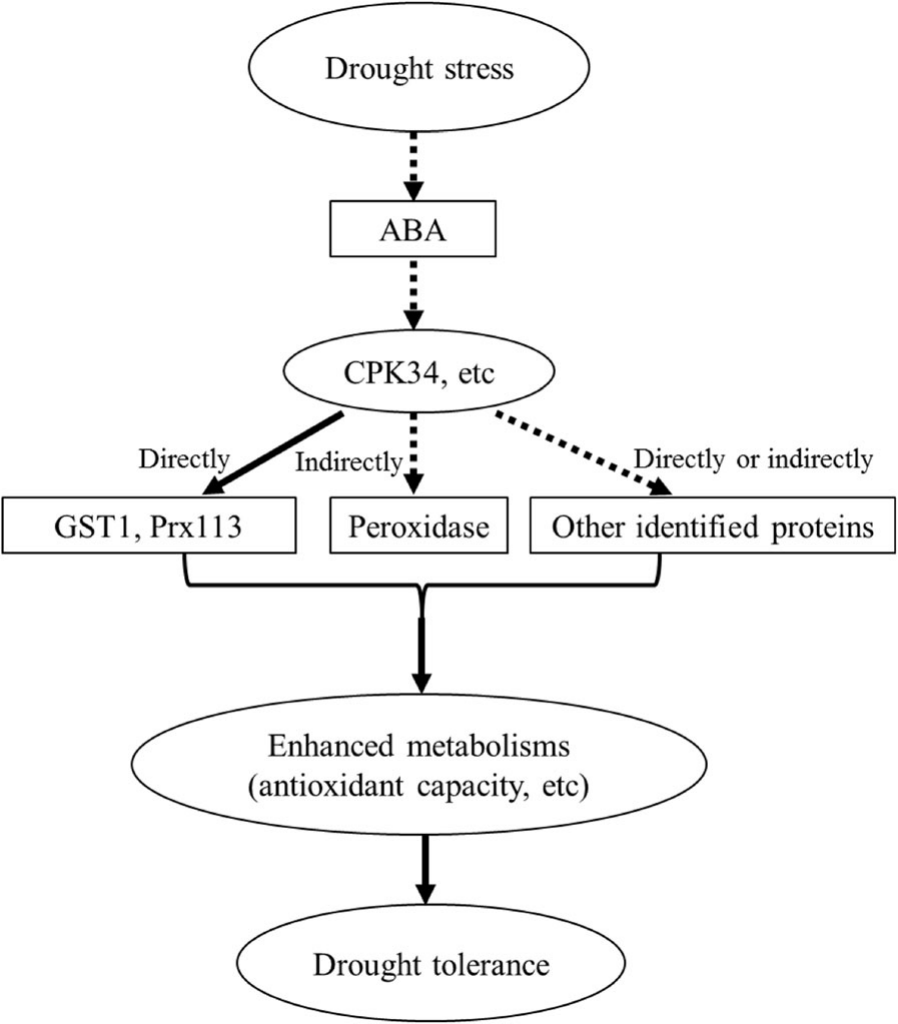
Schematic figure showing signal pathway of TaCPK34 in wheat response to drought stress

ABA is the most critical phytohormone in plant drought stress responses, and several plant CPKs are involved in the mechanisms of drought stress response through an ABA-dependent manner [43]. The *Arabidopsis* plants overexpressing *VaCPK20* gene showed increased drought tolerance, and enhanced the transcripts of *COR47*, *NHX1*, *KIN1*, and *ABF3* genes, which were induced by ABA and drought stresses [44]. In this study, there were ABA-responsive elements (ACGT) of the genes encoding 19 identified proteins (Figure S5). Some of these genes such as dehydrins [45], LEAs [46], prx113, and peroxidase [47], serine/threonine-protein kinase [48], and non specific lipid transfer protein [49], are known to be ABA-responsive, inferring that they could be ABA-dependent manner. Based on these results, we propose that TaCPK34 functions in a signal transduction pathway that is involved in wheat response to drought stress (Fig. 7).

## Conclusion

Our data showed that transcripts of the *TaCPK34* gene were significantly induced by PEG-stimulated drought stress or ABA application, and *TaCPK34-*silenced wheat plants exhibited more sensitive to drought stress, suggesting that TaCPK34 has a positive regulatory role in response to drought stress. Further proteomics analysis and BiFC assay suggested that TaCPK34 protein mediated the wheat drought tolerance by directly regulate the expression of the target genes.

## Methods

### Plant materis and treatment designing

A bread wheat cultivar Zhoumai 18 was grown at the Experimental Farm of Agricultural Faculty at Henan Agricultural University (34°N, 113°E, and 52 m elevation). Some meteorological parameters during different sampling timepoints are listed in Table S1. The grains of Zhoumai 18 wheat cultivar were sterilized with 0.01% HgCl_2_ and fully washed with distilled water. The sterilized grains were grown hydroponically in full-strength Hoagland solution at glass dishes (15-cm diameter) with 50 seedlings each [50]. Germinated grains were transferred into an FPG-300C-30D illumination incubator (Ningbo Laifu Technology Co., Ltd., China) with a 16-h light photoperiod (06:00 ~ 20:00 and 20:00 ~ 06:00, day/night), 25 °C/15 °C day/night, light intensity of 250 μmol m^2^s^− 1^, and relative humidity of 60/75% (day/night). Our previous study found that, two-week-old wheat seedlings with two fully expanded leaves were in the autotrophic stage and were sensitive to abiotic stresses [51]. Two-week-old wheat seedlings were subjected to 20% polyethylene glycol (PEG6000)-stimulated water deficiency, or 100 μM abscisic acid (ABA). The uppermost fully expanded leaves were sampled at 0, 1, 3, and 6 h after treatments.

### Phylogenetic tree construction

ScanProsite (https://prosite.expasy.org/prosite.html) was employed to predicate the conserved domains and motifs of TaCPK34 protein. MEGA 7.0 software was used to construct the phylogenetic tree of wheat, *Arabidopsis* and rice CPKs proteins.

### RNA isolation and PCR or qPCR analysis

RNA extraction and the first strand cDNA synthesis were described in our previous study [52]. Based on the TuCPK34 sequence, a pair of primers was designed to amplify the coding sequence (CDS) of the *TaCPK34* gene using the cDNA from Zhoumai 18. The primer sequences and amplified product are provided in Table S5. Transcripts of the *TaCPK34* gene were measured by using quantitative real-time PCR (qPCR) method following reaction conditions: 95 °C for 5 min (1 cycle), 95 °C for 10 s, 58 ~ 60 °C for 15 s, and finally 72 °C for 20 s (40 cycles). Relative transcripts abundance of the *TaCPK34* gene was calculated using the 2^−ΔΔCt^ method with the wheat *β*-actin gene as the internal control. qPCR primers are listed in Table S5. qPCR was performed using three independent biological replicates with at least three plants in each.

### BSMV experiment

Bread wheat is an allohexaploid species, and therefore, the majority of wheat genes have three copies and these three copies share over 95% sequence identity across coding regions [23]. Because of functional complementation among these copies, it is difficult to explore gene function through single copy-silencing in this species [23]. In the present study, a 219 bp-length conserved cDNA fragment (+ 505 to + 723, the translation start site “ATG” is + 1) from the identified TaCPK34 copy was used to construct its silencing vector (BSMV-VIGS-TaCPK34). Consequently, in this study, all three copies of the *TaCPK34* gene could be silenced simultaneously with the BSMV-VIGS-TaCPK34 vector, and this helped alleviate functional complementation among the three TaCPK34 copies. BSMV-derived GFP-silenced wheat plants were used as the control [53]. Grains of bread wheat cultivar Zhoumai 18 were sown in soil (nutritive soil: vermiculite with 3:2 ratio), and wheat seedlings with two fully expanded leaves and almost identical heights (12 ± 0.2 cm) were inoculated with BSMV-VIGS-TaCPK34 or BSMV-VIGS-GFP virus on the second fully expanded leaves with 10 μL transcript mixtures each leaf, respectively. The BSMV-VIGS method (e.g., viral vector construction, viral RNA transcription, viral inoculation, and TaCPK34-silenced wheat plant measurement) were performed as described in our previous studies [54]. For drought stress, these inoculated seedlings were initially well watered, and then were cultivated in the above illumination incubator without watering for 14 days. Plant fresh and dry weights and relative water contents after water stress were calculated as described previously [55]. And the contents of soluble sugar or malondialdehyde (MDA) in the sampled leaves, which were composed of the inoculated leaves (the second leaves of BSMV-inoculated wheat plants) or the younger fully expanded leaves (the third or fourth leaves of BSMV-inoculated wheat plants) with silencing phenotype after BSMV-inoculated, were determined spectrophotometrically with a UNICO spectrophotometer [UV-2600, UNICO Instruments Co., Ltd., Shanghai, China] as previously described [56, 57]. Experiments were independently repeated three times, and each replicate contained at least five wheat plants.

### iTRAQ proteomics

Total proteins from leaves of BSMV-VIGS-TaCPK34-, or BSMV-VIGS-GFP-inoculated wheat seedlings were extracted at 14 days after water stress using the trichloroacetic acid/acetone method. Four biological replicates were performed and each replicate with at least six plants. iTRAQ proteomics assay wasperformedas described in our previous study [52]. Samples were labeled with 113–116 for BSMV-VIGS-TaCPK34-inoculated wheat seedlings, and 117–119 and 121 for BSMV-VIGS-GFP-inoculated wheat seedlings. The MS/MS spectra were searched against the NCBInr database (https://www.ncbi.nlm.nih.gov/taxonomy/?term=Triticeae, version 10/06/2018, 192,314 entries) to identify the proteins, which were isolated by using MASCOT software (Matrix Science, London, UK; version 2.2). The quantified peptides were calculated by using the ProGroup algorithm (AB SCIEX). Then the identified proteins were screen using several criteria (i) the cRAP database (ftp://ftp.thegpm.org/fasta/cRAP) was used for filtering the identified protein species; (ii) the confidence and the false discovery rate (FDR) of proteins were ≥ 95% and < 0.05, respectively; (iii) the proteins were commonly identified in four biological replicates; and (iv) multiple testing corrections (*t <* 0.05) were used to adjust *P* values for controlling the FDR, and ratios were further analyzed by using Benjamini and Hochberg (BH) test. Proteins whose abundances differed significantly by fold-changes of ≥1.5 or ≤ 0.67 between BSMV-VIGS-TaCPK34- and BSMV-VIGS-GFP-inoculated wheat plants were selected and considered to be used for further study.

### BiFC assay

The interactions between TaCPK34 and the identified proteins were tested by using the BiFC method as described previously [58]. The CDS regions of the genes encoding wheat GST1, Prx113, and peroxidase identified in our proteomic data, were amplified and ligated into the BiFC vector, which contains a yellow fluorescent protein in carboxy-terminal part, to construct a YFP-C-genes (YC-gene). Likewise, the *TaCPK34* gene was also inserted into the amino-terminal part of the yellow fluorescent protein (YFPN) to form YN-TaCPK34. The proteins of HAL3 and Di19, which were confirmed to interact with plant CPKs in the cytoplasm and nucleus [38, 58], respectively, were used as positive controls in this study. Then, the transient assays of interactions between TaCPK34 and other proteins were performed by using the epidermal leaf cells of tobacco, and the fluorescence of YFP was observed with confocal laser scanning microscopy (LSM710, Karl Zeiss, Jena, Germany).

### Statistical analysis

All experiments were independently repeated at least three times and each sampling was analyzed separately. SPSS 20.0 software was used for statistical analyses and statistically significant differences were measured by using Duncan’s tests at *P* < 0.05 level.

## Supplementary information

**Additional file 1: Supplemental Figure S1.** Amino acid sequence alignment between TuCPK34 (EMS58754) in *Triticum urartu* and TaCPK34 in *Chinese Spring* (CS-TaCPK34). **Supplemental Figure S2.** Abundance of TaCPK34 protein in wheat grains after anthesis. **Supplemental Figure S3.** The location of silenced gene-specific fragment in full-length of the *TaCPK34* gene. **Supplemental Figure S4.** BSMV-VIGS-mediated silencing of the *TaCPK34* gene. **Supplemental Figure S5.** The promoter sequences of the genes encoding the identified proteins with known functions.

**Additional file 2: Supplemental Table S1.** Meteorological parameters (rainfall, temperature, etc.) after anthesis in the experimental field.

**Additional file 3: Supplemental Table S2.** The selected CPKs from *Arabidopsis thaliana*, rice and bread wheat for the phylogenetic tree analysis.

**Additional file 4: Supplemental Table S3.** All peptides of the identified proteins in leaves of BSMV-VIGS-TaCPK34- or BSMV-VIGS-GFP-inoculated wheat seedlings suffering from drought for 14 days.

**Additional file 5: Supplemental Table S4.** All identified proteins in leaves of BSMV-VIGS-TaCPK34- or BSMV-VIGS-GFP-inoculated wheat seedlings suffering from drought for 14 days in all four biological replicates.

**Additional file 6: Supplemental Table S5.** Primer sequences used in this study.

## Data Availability

The mass spectrometry proteomics data used in this study have been deposited in ProteomeXchange Consortium with the dataset identifier PXD008567 (http://www.ebi.ac.uk/pride/archive/projects/PXD008567) and FTP Download: ftp://ftp.pride.ebi.ac.uk/pride/data/archive/2020/08/PXD008567. Others data used and analyzed during the current study are available in the manuscript and its additional files.

## Abbreviations

ABA: Abscisic acid
BSMV-VIGS: Barley stripe mosaic virus-induced silencing
CaMs: Calmodulins
CBLs: CaM-like proteins and calcineurin B-like proteins
CDS: Coding sequence
CPKs: Calcium-dependent protein kinases
iTRAQ: Isobaric tags for relative and absolute quantitation
IWGSC: International Wheat Genome Sequencing Consortium
MDA: Malondialdehyde
qPCR: Quantitative real-time PCR
