# Supplementary material for: Functional characterization and regulatory mechanism of wheat CPK34 kinase in response to drought stress
Source: BMC Genomics. 2020 Aug 24;21:577. doi: 10.1186/s12864-020-06985-1 (PMC7444251; doi:10.1186/s12864-020-06985-1)
Supplement: Supplementary file 1 — Additional file 1: Supplemental Figure S1. Amino acid sequence alignment between TuCPK34 (EMS58754) in Triticum urartu and TaCPK34 in Chinese Spring (CS-TaCPK34). Supplemental Figure S2. Abundance of TaCPK34 protein in wheat grains after anthesis. Supplemental Figure S3. The location of silenced gene-specific fragment in full-length of the TaCPK34 gene. Supplemental Figure S4. BSMV-VIGS-mediated silencing of the TaCPK34 gene. Supplemental Figure S5. The promoter sequences of the genes encoding the identified proteins with known functions. [file 12864_2020_6985_MOESM1_ESM.pdf]

```
TuCPK34                                                MALKFLRSRMLMFLMEDHTGVIKY.....RQTMI   29
CS-TaCPK34 MGNCCAGSGDAEPEAAADPSTRRAGASVQAGGASPSSAPAQNKPPAAIGPVLGRPMEDVRSIYTVGKELGRGQFG   75

TuCPK34    VIEEKYILATGQKFACKTIAKRKLSTKEDVEDVRREVQIMYHLAGQPNIVELKGAYEDKQSVHLVMELCAGGELF  104
CS-TaCPK34 ITSLCTHKATGQKFACKTIAKRKLSTKEDVEDVRREVQIMYHLAGQPNIVELKGAYEDKQSVHLVMELCAGGELF  150

TuCPK34    DRIIAKGKYTERAAAALLRTIVEIIHTCHSLGVIHRDLKPENFLLLSKEEDAPLKATDFGLSVFYKQGTHACIYL  179
CS-TaCPK34 DRIIAKGKYTERAAAALLRTIVEIIHTCHSLGVIHRDLKPENFLLLSKEEDAPLKATDFGLSVFYKQGE...VFK  222

TuCPK34    LLLSRPSKIANETASFRCSRKNSGAIVGCRGGVQGHRGQRLLHRAGESEHGIFNSILRGQIDFSSDPWPRISSGA  254
CS-TaCPK34 DIVGSAYYIAPEVLKRNYGPEADIWSVGVIVYILLCG...VPPFWAESEHGIFNSILRGQIDFSSDPWPRISPGA  294

TuCPK34    KDLVRKMLNSDPKKRISAYDVLNHPWIKEDGEAPDTPLDNAVMNRLKQFKAMNQFKKAALRVIAGCLSEEEIRGL  329
CS-TaCPK34 KDLVRKMLNSDPKKRISAYDVLNHPWIKEDGEAPDTPLDNAVMNRLKQFKAMNQFKKAALRVIAGCLSEEEIRGL  369

TuCPK34    KEMFKSMDSDNSGTITVDELRKGLGKQGTKLTEAEVEQLMEAADADGSGTIDYDEFITATMHMNRMDREEHLYTA  404
CS-TaCPK34 KEMFKSMDSDNSGTITVDELRKGLGKQGTKLTEAEVEQLMEAADADGSGTIDYEEFITATMHMNRMDREEHLYTA  444

TuCPK34    FQYFDKDNSGYISKEELEQALREKGLLDGRDMSEIVSEVDADNDGRIDYSEFVAMMRKGAPEGANPKKRRDVVL   478
CS-TaCPK34 FQYFDKDNSGYISKEELEQALREKGLLDGRDMNEIVSEVDADNDGRIDYSEFVAMMRKGAPEGANPKKRRDVML   518
```

Supplemental Figure S1. Amino acid sequence alignment between TuCPK34 (EMS58754) in *Triticum urartu* and TaCPK34 in *Chinese Spring* (CS-TaCPK34).

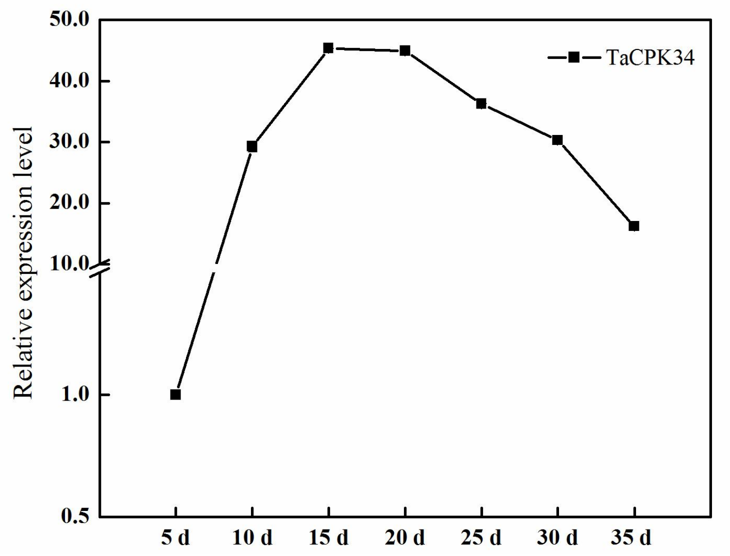

Supplemental Figure S2. Abundance of TaCPK34 protein in wheat grains after anthesis. Notes: expression level of TaCPK34 protein at 5 day after anthesis is acted as control (value=1.0) to calculate its expression levels at other sampling timepoints (10, 15, 20, 25, 30, and 35 days after anthesis). Each value is the mean ± standard deviation of at least three independent measurements.

```
TaCPK34-3AL   GACCGGATCATCGCCAAGGGCAAGTACACGGAGCGCGCCGCCGCGGCCCTGCTCCGCACCATCGTCGAGATCATC   525
TaCPK34-3B    GACCGGATCATCGCCAAGGGCAAATACACGGAGCGCGCCGCCGCAGCCCTGCTCCGCACCATCGTCGAGATCATC   525
TaCPK34-3DL   GACCGGATCATCGCCAAGGGCAAGTACACGGAGCGCGCCGCCGCATCCCTGCTCCGCACCATCGTCGAGATCATC   525

TaCPK34-3AL   CACACCTGCCACTCCCTCGGCGTCATCCACCGCGACCTCAAGCCCGAGAACTTCCTCCTGCTCAGCAAGGAGGAG   600
TaCPK34-3B    CACACCTGCCACTCCCTCGGCGTCATCCACCGCGACCTCAAGCCCGAGAACTTCCTCCTGCTCAGCAAGGAGGAG   600
TaCPK34-3DL   CACACCTGCCACTCCCTCGGCGTCATCCACCGCGACCTCAAGCCCGAGAACTTCCTCCTGCTCAGCAAGGAGGAG   600

TaCPK34-3AL   GACGCGCCGCTCAAGGCCACCGACTTCGGGCTATCCGTCTTCTACAAGCAAGGGGAGGTGTTCAAGGACATCGTG   675
TaCPK34-3B    GACGCGCCGCTCAAGGCCACCGACTTCGGGCTATCCGTCTTCTACAAGCAAGGGGAGGTGTTCAAGGACATCGTG   675
TaCPK34-3DL   GACGCGCCGCTCAAGGCCACCGACTTCGGGCTATCCGTCTTCTACAAGCAAGGGGAGGTGTTCAAGGACATCGTG   675

TaCPK34-3AL   GGCAGCGCCTACTACATCGCGCCGGAGGTCCTGAAGCGGAACTACGGGCCGGAGGCGGACATCTGGAGCGTCGGC   750
TaCPK34-3B    GGCAGCGCCTACTACATCGCGCCGGAGGTCCTGAAGCGGAACTACGGGCCGGAGGCGGACATATGGAGCGTCGGC   750
TaCPK34-3DL   GGCAGCGCCTACTACATCGCGCCGGAGGTCCTGAAGCGGAACTACGGGCCGGAGGCGGACATATGGAGCGTCGGC   750
```

Supplemental Figure S3. The location of silenced gene-specific fragment in full-length of the *TaCPK34* gene. Notes: Length of the selected fragment is 219 bp (from +505 to +723, the translation start site “ATG” is +1).

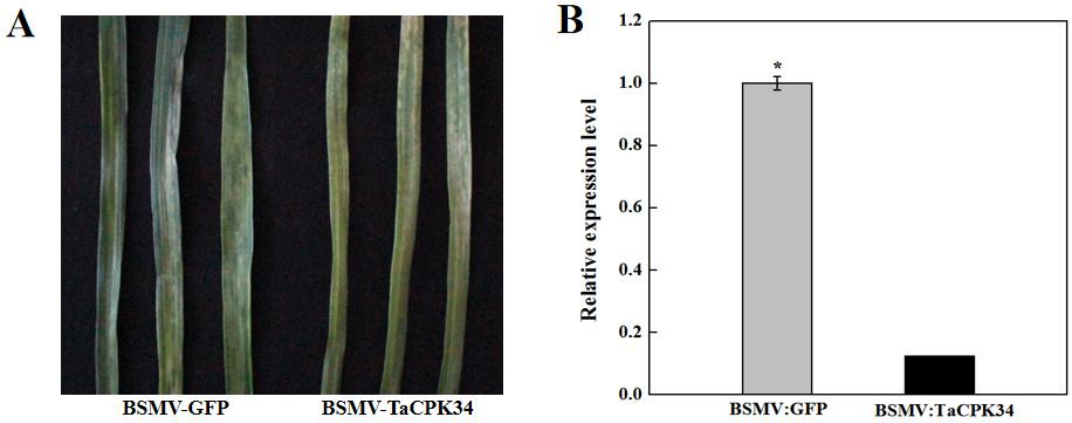

Supplemental Figure S4. BSMV-VIGS-mediated silencing of the *TaCPK34* gene. A, Phenotype of the fourth leaves of BSMV-VIGS-TaCPK34- or BSMV-VIGS-GFP-inoculated wheat seedlings; B, Transcripts of the *TaCPK34* gene in leaves of these two treatments. Transcripts were determined by qPCR using the *TaActin* gene as the internal control. Each value is the mean ± standard deviation of at least three independent measurements. Asterisks indicate statistically significant differences ($P < 0.05$).

Dehydrin DHN2 (NCBI accession no. XP_020155660.1; IWGSC accession no. TraesCS5A02G369800.1)

AGGCGGTGTTGTGGATCCGGTGACAGTAGTAGATTGGGATTCTTCCTTGTAATTAGGGTTGCTAAGTCGCGGGCGCATTGACCATGGCTATG
GTGGCGGCGACACTCCATGTCCTGGTCCGGGCTAGCAAGGCAGGGGCGATGTCCCCGAGCCAGAATAATGTCATTCTCGCCTTATCTTCGCA
CCAGTGGTGCGTTTAGCACCCTCGAAGGG**CACGTG**AAGGTGTGTCTCAAACCTAGATCCAAACATATACACATTCTCACAAGTTGATAATAT
TTCAAACATATAAAGGACCAAAAGAAATTAACGTAATTCTTAGATGAAACAGACGTTGCAAGCATGTCTTTGGTTTGATCATTGTAAACGTC
GAGCATGTTGACCAAAATGTTGTATGTGACACTCTCTCTCCCCTCTCCTCTGCAACATGGCTGCCGGATGCTTTCCGTGCCAACTCATTTGC
TCTTTAGTTTCATGTCTAGATGTACAAGCAATAATGCAATACATTTATCAAGATAAATATTAAGTTGAGTAAATGCATCGTTGTTTAATGGA
AGGATGGATGTAAATGCATTGACCTTCCCCGATAATATATCTTGTATTCGGAATGTTGGCGGCACTAGCTTAGCCATTTAAAAACATTATCT
ATCTAAATTATCTTTAAAATACAAATAATCCATCAATAAATCAGAATGTGCCAACCAACAGTATATATAGAGACAACATTGATAACGAGTTA
TGAGCTTCGATCTACAAAGATTGCATGGAGATAGAGTTGATGGAGATGGTTTTATGGCACCAATTATGTCCACAAGTGTTCATAGTATATGA
TGAATTTAGCATCTCTATTTCCACATTTCTGTCAACCTTTTCCTTTCTCACTCTATTTTTGGAGACATATTTTATGATGGAAGGTGGAGTGT
CGATTTCCGCTTCAGCAGCACTTGATTGCGCTTGTAGAGTGTGCATGATAAGACATAGATCACTAGTGGTGGAGCTTCATATGTTAGAGATA
CGACACACAATCGAACACGATGAAGAACGAAAACAAGCAAGGGATACGGGATTTTAATGTGGAAACCCCCTTTAACACGAACGAGAAAAAAC
CAAGGACGCCAGCCAGACAAACTTCACTGTATCAGAGGAGGTTTAAAACGTCAGGAATTTACAGCCGATCAATTATCCTATGCAGTGGCTTA
CAAAACCCACCCCCCTCCCCCCCCACCCCCACCCAAGCGTCCCTGTCGGGGGCAGCCCACATGGGTTAACTTTAGCCCAAGCCTACCAGCGC
ACCGCTCGCTCTTCAAAAGCTAGCCTTTTCTCTTTATACTTGATGTCTTCCAAGAGGCAAAAGTTATATCGGATGCCAGGGGCAGAGGCCAA
CGGTTAAGACTGGGTAGGCCACGGACGAGGAACCGTTGCGCACAAAGGAAGAAGGAAGAGAGGAAGAAGATGTACACTGGTCTGTCTCTAGC
CGTTGGAAGAAGATCCAGCGGTCCCCAAAAAAAGGACCTACGCAAAAGATATTTTCAAAGATAACGGGCGCTTGAGTAGAACACATATTTTC
CACGTTATCACCCACGCTGTCGCTAACGCAATAATACTAGACGTACATGTGATTGATACAAAACTTTACAGGTTGAGAACGTGGCCTCATCT
AATTGGCCCCCTGATTTTCATGGATGGGCCCACCCTTCTAATTAAATCTCTGCCAATCACAGCCCATCAGCCCTATAAACTCCCTGTAACTT
CGTGTACGTGTAGCATTGCTCTCGCTAACGTGTACCACTACTATTCATGCATATGCATGCGTAGATGGAGGAGTCATGCCCTTCGAGAGAGT
GCGAAGCCCTACGTGCCGCCGCCGACGAGCCCGAGTGGCTTTGTATATTGGCGGGATGACCACTCCACGCACTTGTACTGGTGTCAGTGTGC
TTCTCGAAGTCGAACACCTGGACCATTCTGAGACCTATAAATAGCCACCTGCAGTCGCACTTGGCTGCATCGCAACCCGCAGCAGTTCAACA
CTTACACAGCCATACACATCACCTGTAGATCGACCGGTGTCTTACGCGACG**ATG**

LEA3 protein (NCBI accession no. ALD18912.1; IWGSC accession no. TraesCS1B02G381200.1)

GAAAACACAAACATTGTTTAAAATATTTTTGATACTCCCGAACAAAATTTGAAATATGTGAACACATTTTTGAAATGGGAACATTTTACGAA
ACTCGCGAACAAAAATTTAAAACACAAACATTTTTTTGAAAATTGTGAACAAATTTTGAAATAGGAACATTTGTTGAAACTCACAAACAAAA
ATGAAAATATGAACATTTTTATTAAATATGCAAACAAATTTTGCAAAGTTGTGAAATTTTGAAAAAAACATAAAGAAAAGAAACAGAAACAG
AAAGTAAAAAAGGTAATAAACAGAAAAGGAAAAAAAGAAATAGAAAACAATAGAAAGCCGAAAAGAACCAGAGAAAACGATACAGGAAAAAC
CCGGTTCAGGGAACCTTCTAGAAGGTTCCCAAAACCGGTTGAGTGGGCCGGCCCATGTCTTTGGCTCGCTGCGCTCCTATGTGCGGTTCTCT
GTGTGATTGCTCGACAGTTCGCCGCAGCGAGGCGCGAATAGGATTTTCCGTGTTGCGGCCGGGAAATATGTCTCACATACTGTGCTCGCTTC
TGGAGCTCCTCTAAACGGGCTGGCCCATCATTGTAGATCATGTGCGTTTTTTTTCGGGAAGGTTCTGTGGTCTGGTTCAGACCTGTTTTAGA
ACCCTCATTGTGTTTTCTTCTTCTTTCATTTATATGTGTTTTTATCGGATCCTTTACTTCACAGTTTGTTTCATTTTTTTCAACACATATCT
CTTTTTCCAATACACATTGTACATATTCAGAATACATGATGTACGTTTTCCAAATGCATGTTTTGAAAAGCTTTTGAATACATCTTAAACAT
TGTTTGATACACTTTGAAAATAATTTTAATGGCATTAATCATTTTCTAAACTACGCAAGATATTTTTTTAATGTCAGAACATTTTAGAAAAC
ACGGGAACATTCATTTAAATATGAAGTATATTTGTTTGAATGATATGAAACATTTTGTATTTACATGGTATGAACATTTTCTAAAATATGGC
ACACATTTTATGAATGATATCAACATTTTATAAAAAGTTGTGCAAATAGTTTTTTATACTGCATGAATATTTTCATGTGGGATGAACATTTT
AAAAAATTGAAGTAAATTTTTTTATTTGGAAGACGCAAGTAAAAAAATAATACGATCCGCTTGGAAGCTAGGGCTCTTCTTGAGCAGGGCCA
CTTCATCTAGGCCATTTCGGACATCCTTGAGACGGAGGGTTCTTTCATCTCACTCACAGCTAGACAAAGACGCGCCAATGCCCCCGTAAAAA
AAAAAAAAAGATGCGCCAATGCGTTGAGCTTCAAAATATTTTTCTTTCTTGGTTATAAAACGCGAGATTTCCGAGCGAACGTACGGTGCAAT
TTTCTACCCGCTGTCACCGCTGCTACTTCTGGATGAAGAAGCGTCCGATCTCATCGAGTGGAGCGACGTTGCGTTGTTTATTCGCGTTGCTT
CCTTTTGCGGTAAGACAGGCATCG**CACGTG**ACACCGCGCGCACACAAACAAATTACACCGAGAGAAAAAACAACACGTCGATGGATCATCAG
AGATGCCGATGCACATGGCGGCGACGATGGCGTCGATGCATGCTCCACCGCACCGCCACCAAGTTGCAACCTCGCAGCCAGAGAGCCGCATC
CAACTTGTGCTCGCTCGCCTGCGCCACGCCTTTACACTTGTTTATTGCAGCTTCTTCGCCCCTTTTGGCCTCTTCTTCCTCCGGCATGGGCT
CGATCGAATCGGTTCCATCTACATGGCAGAGCTTCGCGAAGGTACGGCTGGGGAGCGGCAACGCGTGTCCTCCCTACGTGGCGGCCATGTAC
GAGCACCGCCGCGCAACGTGGCTCATAGCTTCTGCCTACAAAGGCCACCTGCTCCGTGCTCCTCACCATATCACAAGAAGCCGATCCATTCC
AAGCACAGTCGAGCAGAAGCCTAAAGCGAGTGACATTTCCCCCTTCCGAGCTAGCTCAAGAAACCAAT**ATG**

Low-temperature-induced 65 kDa protein-like isoform X1 (NCBI accession no. XP_020162307.1; IWGSC accession no. TraesCS1D02G163800.2)

GGCGACTTGGAGAAAGGGAAAATGGTGGTGCATGATAGCCTCCCGGTTATACACACAAGGGAAGTGCCCAAGCCGGAGTTGCACTGGACCCC
TCCTCCACCGGGGTAGGCTAAGCTCAATACAGATGGCAGCCATGTCCCTCCAACCAGAGAGGCGGGGGTGCTATGATTCTTCGTGATGATAG
AGGCAACATCATCTATACTGCTTGTAGACAGCTCCTTACGTGCGATAATGGCCTTGAGGCAGAGCTGGCTGCGTGCAGAGAGGGCCTGGAGC
TTGCGCTTTACCGTACGAACCTGCTTATTATGGTTGAGCTCGACTGTGCAGAAGCGATGTCTATGCTGCTAGCACGTACTGGAGATAGATCG
CAGTACCGTGTGCTCATTGAGGAGATTCGAAGACTAGCACAAGATGCTCGTAGGGAGATTTTTTTACTCTTATTAGTCGCTCGCAGAATAA
GGTTAGTCATGCGCTTGCCGCGTATGGACGTGCTACACCCTGCACTGTAGTTTGGCTGTGCTCGGGGCTGGACTTTGTTGTAAACCTTTCGA
AGGCTGATATGCCTCCTTGAGTAATGAAATCTCCTTTTACCCCGAAAAAAAACACGCGCGCACTGTCAGCAGATGGAAGCCGGCGGCGTCCA
TGTCCACGGGGCTGCTTTCGATCCAGGGCAGGCGGCCACCATGTCCTGTCCATGTCCAGCACATCACAGCACGCACATGGCCAAGCACGTAT
CGTCAAACGCTGGCGCGCTCGCGGCCGACGCCGGCGACTGCATATGCCTGCTCGAACTCCGCTGCTGCATCCAAGCTGTTCGACGAAATGCC
CGTCGCTGCTGCTGGATCGATGCATGCACGTACGCATCACTGCTGAATCGATGCAGTCCGCCGAGACGCAAGTACCCATACAAAGAAAGGAA
AGAAAAAGACATACGTACAACGGTACAAGCTAGCTTCAAACCTGCAGTGCTGATTGCTTCCGGCTGGATACGAAAGCAACTGGAGCTAGCTT
GCGTGGGAGCAGTTTCATCGCACTCGTCGGAGTATGCAGTGCAGGCGCGCGTGCGACTGCGTAGGGGAGGAACAAGCGCATCGAGTACCAGC
ATCGCTATGGGCATCCTATTGCATAATATGGAATACAAATATATCTTCCGACAGATAGAGCGGGATGGTCCGGCGGTCAGCTCGGTTTGCTG
CTCGAACCCTCGTCGGCGACTTATATTTTTTGCCTGAAACCCTTCAGTATTTCTTTCTTCAGCACACTGACAAGACAGGACCCACATATGTG
TACACCGACATGGCCATAATTAATTTCGTCAAAGTCAATAATCCCGGACTCCTTGTGCCACATCACCAAATCCCCTCTTAAATCGGAGTGAA
GGTTCATTTAGAGCGGGATCAAAGAAAACAGGGTACAAACTTCAGAGATTTGGACGTGAGGGTCTGTTTTAAACGAGATCTATAACATTAAG

GTTTAGATCAGACTTGACAATATGCTTTGTGTAAGTTGCATGTCATATCTTATTGCTCTAATATTTGGTCAAAGTTAGCCTCGAAAAATGCA
TTAGGCCTACAGACGAAAGGAGGGAGTAAACCTGTTAAGAGTAACCTGACACCCTTCAATTATTTGGTCGCTGTATTACCGAATGTGTTTTC
TTGCCCATCGAAACTGTTCTCATAAATTCTTTTCAAAACACCGACCAAAGAGGCAAAACCCGACGCGGCGCATCCCGGGCCAAAAGGCGGAC
GCCACATGACGAGACGGCTCGTCGGCCTCCATTCCACCTCGTCGTAAACGCGGAACCGCTCCATGATCACCCCGGCCAATCAGGGATAGCTG
GACCTTCCAGTCGCTTCCACCCCCCAAACTAAACCCCGTCACGGCACGGAGCGGCAGCGCCACGCGTCCCTCCTCTTCTCCACCCACCC**CAC**
GTGTGCGTCGAGGTGGCGCTCGCCTCCCCGTTCTGCTCCGCTTATATCCCATCCTCAGTCGACCAAGGCTTTCACCATCCCACACACAGCAT
TTAGCGATCGAGCAACAAGCCAATTTCGTCACAGTGATCAAAGAGTCTGAGATCGCCTAGTAGCTGCACCGACGTACGTGCGACG**ATG**

Barwin-like (NCBI accession no. XP_020147546.1; IWGSC accession no. TraesCS2B02G581900.1)

AGACATATTTTTGCTCATTTGCATGGTAAACACTTCTTACAGACTAAACAACTGATGCCCCGAATGCTCCACCTACCCGCAAACATGACGAC
CAACGCACCTTCATCATGGCATAAAGACATCTCTATGCAGAAGCCACCAACAGCGTCACTAATAGATAACAATGAGCTGGATCTGGTCGAAC
TAGTAATATCTGGTCCCGGTCGACGCTGCAGCAACCAAATGTCAGCAAACAGCTAGCTCGTGGGGACGAATCTCCAGAGAGCCAAAATGGGG
GCACCCACCCCGAATAATTGGGATGAGCTGCGGGGCAGGAGCACCGGAGCTTGGCGCCGACGTTGTTGCGTCTTCCACCTGCTGGAGACACA
AACAAGGGAACAATTGCACCATCGGCAGTTGTTGCACCGGGCAGGAGGGGGCATGCCTTCCCCACCGCGAGCACCTACCGCTGCCATCAACC
GCGTGGGCTTAGCCCAACGACTTCCTCCGATGACAGTGATGAGAGAAGGGAGGCAAGGAACTGGCCTGGCAACTCGATCAAGGGTATGCCTA
TGTCGCTTAAGAGGCCATGAGGGAAACCAGTCAGGGGGTAATTAAGGGTTTAGATCTTACAATATAAAGTTGAAGACGATTTGTGGCACCCA
GGGACGGGAGACGAGTGCATGTAGCCACATGATTGCATCAAGATCCGAAAAGTGGTTTGTGGTTTGAGCTTAATTGTTGCATGCATGCAACT
TTTTGGTACAACTTTTGGCATAGGCCACATGCCCCATCCATCCCACCTCCAATGCTTTTCATTTGTACTTGTAAATTTGAATCTATTACATC
TTTTGAATCAAAAGTTCAATTTATATTTCGTTTGCATATTCATGTCCCTTGTGTCGAAAGCTTTGAAACAAGCCCCTTTTGAATACGTTTTG
ATAAATTCTAAAACTTAAGTGAGTTTCAACTACTAAAACTTAATAGAATGAATGATAAATTTAGCAAGTTTCAAAGACTAAAACTGAAACCC
TAAGCCCTAAGCCGGAAGTCCTAAACCGTATACCCCTATCAAATGTAACTTGGTGAAACTTATGATGATTCTCTAGTTTTAATATTTGAAAC
TTAATAAAATAAAATAAAAAGATATATAAATGTATTTAAAATTAATTTTGTTCAAAAGGACTTGTTGCAACGAACACGAATATGCAAACGAA
ACTTAATTTAGACTTTTGGCTCAGAAGATACAATAGATTTAAATTTTGAGATAAAACGAAAACACATGAGAGGTGGGGTGGGTGACTCATGC
AGCTCTGCCAAAAGCTGGCAAGCATGGACCCCAGTACGGCGATTAAGAGCTAATGACATAAACTATCTAGTAAACCAATCTTAAAGCAAATG
GGTTGCCGCATGCCTTTCTGCCCTGGTGGATTTTTCGATGTGAAGTGTGGGTATACAAAAAATTGCAGCGGAAGCATCTATATACTATATAA
TTAATTGTAATCTAATTGTTGTGATTATATGTCGAGTGAAATTATGAAGATGTGAGGTCCGGTTTTGTTTTTGCGTTTTTTCTTTGGACTTG
TTCTAGGCAGTTGGTGTTGTCGGTATCTCAATGAATTTGTGAGAACTCTATCTCATAACAATAAATCAAGCTATCTTATCTAAAACTTCAAT
TATGCGCAAAGATATATCTTCCCTCTAGCTTTCGAC**CACGTG**TTTGCAATTGCAGCCGCATAAATTTGTAGAAAGTGACACGCTGACATGAT
TACATAGGCATCCATCCACACTTGTAACAATTTTGCTAAATTTTCCAGCTAGGTAGCAGGA**CACGTG**TGCCACAGCCCTTAAAAGCATATCT
GCACATGTCCTTATGAGCTGTTCATGCAATTGATTCCTAGTTTGTAGAAAATTCGTGGAGGCTGCGAAGAGAAGAAAAGTCTACCATCACCG
TATCATATATTAATTTCCAGCGTTCCATTCCACCTCTATATAATAAATGGTAGTGGCGAAGAGCGACTCCCATTCGAGTGTCAGCACCTGCT
AGCTACCACTCCTCACATCCTCTGGTACGATCACCCTCCTCTGCTGCGTTGACGAAG**ATG**

11 kDa late embryogenesis abundant protein-like (NCBI accession no. XP_020190115.1; IWGSC accession no. TraesCS5D02G177300.1)

TGCGAGATGGCGATTGGATGGTGCTGACACATCTTGCTAGCGAGGAGGCTGTTTGCCGAAATTGGACTTTGGATTGTTATAAAATTTCAGTT
TAGACCAATTCTTGTTAAACATAACATGTTATTGTGACAACTATTATGAAAAAAATTGATTTAGATAATCAAAATGATGAAGAAATTCCTAA
ACTTACGTGCGTTG**CACGTG**CACCCTTTCCAATTTGAAGGGCTGCTTATCGGTTTTAAATCTTGAAGTTTGCACGGTCACTAATATGACCAT
GCTATTTTAGAGTGATAACCTTCATTTCAGGCAGTAATGAAACTTGAAATGCCAGTTGACTAACAGTAATAAACACCAAGAATACTAGTTGA
TGTTAATGGGAATTATGATTAGCGATGATTCATTTTATGCTCTGGTGGAACGGGTCAAGCCGGTTTGCGCGGTGAGTATTGTCCGTTACTTA
TTTTCATACTAACTATATATATTGTTTGTGCAGGATGGATATGAGTTGAGAAAGTAGTTTTTATTATACTTGCTAAAACATCGTGGGAATGA
AGCTAAAGACAACTTTCCGTATATTGTGAAAGAATTTCTTAAGCGTATAACCTAGATATTAATAATTTTATAATAGATGTTTAGTTGAAACT
CCATTAAGTTTGTTACAAGGACATGCCGTTAATTTTTTCTTTTGGCAATTTACATTGCAAAAATGTACTTTAATTATTAAATAATTGTGTAT
GTGTTATATTGTCTGTACGTATCAAATTTAGCATGCATTATCTTTATATAACTCTTGCAAACTATTTCAAGAGCCCGTGGCAACGCACGGGC
ATTGTACTAGTACTAGTATTGTCCAGCATTTCTTTACTGCAAATACCGGTAAGGCTACTAGTCGATGATTGAGAAAGAAGTTGGCCTAAGAG
CAACTCCACTCGTTCGGCCCCCAGCGCAGCGGCCGGCGGTATTTCAGACCTTCGCCCGGCGCTTTTTAGGCCCTGGGGGCGATCTAGGTCCC
AGCCACGCCCCAGAGGCCGACCCCAAGACGTTTAAATTCAAACCTGACATTTCCCGCTACCCAAAAAAAGCCACAAGTCCGGCGATCACCAT
GCCACAGTTCGGCGATCCGACGCTTTGCTGGTTCCAGGGAGAGCGTGCGGGCCGTGGCCGCTGGGCGGATTCATCATCCCCGCGCGAGACGC
CTCTGCTTGTTCCTCCACGCGATCTGCCTGCTCCGCCACGGCCGCCGCCGCCCCCGCCCTGTCCCAGGCCTTCTTGGCGTTGAGCCTGTTCT
GCCGGTCGGTGGTACAGCCTCCCGGCGCTGAACCTCTGCCTTCCAGTCGGCGTTTGACATGCCCGGGGGCTTGGACGGCGGTGCCCTCTGCT
TCGGCTGGGTGGCGTTGGCATCGGTAGGGGCCCGAGGGGCCGTGTACTTCTTCGGCGGCATGATGTCCGGATGAAGGGGAGAGGGAGGAGAA
TGGCGGGAGAAGGGGGAAATGGAGGGAAACGAAGGGGGAAAAGCAGGGGGGATCGGCGGGAAAAGGGCCTTCTCTCGCCGACAGAGCGGACC
CACGAGCCTTTTCGCTTCAGGCGGCGCCCCCAGGCGACCTCCGGGGGCTTGGGTTCGACTCGGGTTCGCCAGCTATTATTTTGGCCCGGCGA
TAAATGAGATTATGGGACGCGACTGGACCGATTTTTGGGCACCGGCGTTAAAAAGTAGCCTCTTCTCCGGCTGGAGATGCTCTAAGTTGAAG
CTTCTCGTCCTCCTAACCAAAACGAGCCAACTCAAAGAAAACGAAAAATAATCGAGTTGCTGTTACTTTGAGGAATTGATTCGGGTTCTAGA
GGACACGCGTGGAGTGCCACAGGCGCCGGCAGCAGCTTCTCCGTCCGAGTA**CACGTG**CCCCTCGCCGACCTGCCGACCTGGGACACGTCCCC
CCGTCGGCGGTACGCATCAGCGCTTCACATTCATCCACATATATATATACTCTACGGAGTACGTAGTTACCAGCGTAGAACCGCATCGAAGT
GAAGACAAGAAACACAAGAGATCGACCGACACCGAGCCACAGGAGACCGTGTAGGTTTGATTTTAGCGAGCCAGAGGGAGGG**ATG**

LEA 3 (NCBI accession no. ALD18913.1; IWGSC accession no. TraesCS1D02G369200.1)

TATCAACAGAGCAATTAAAGTGTCATTCATATTCATGTACATCAGACAAGGGTATTTATAACTAAAATAGCACCTCACATTAAGATCAGTCA
ACTGCTCTTAAACTAAAAAATTAGGGATTATTATGTAAAGTTGATCCAAGCAAAGCACTCACCGAGCATGAAATCCACATCTCTAGCAGCCT
CCTACTTCAATCCATGTACTCAGTTGTTGGCTTGTCTTGCAGGCACCACAACCCTATCAGCAAACAGTGAGAGGGAGAACATAAATAAATCA
AACAACAGAAAAATTCCTCAGATCGGTACAATGTTGTATTAACAGCGCAACACATCAACTTTGAATTTATCTTGTGTTTCTTGTCTTCGATG
CCAGCTCTGCTGACTAAGATTGTACAATGAAAGAGCAGAAAAATGATCTTAGTTGCGCACACAAAAAACATTTGTTGGGTGAAGGAACTTCG
TTTGGTGAGTATAGTTACATCTAATAAACAATATCTTCTTAAGAACCAACACCATATTTCTACATACAAAAGTGAAAAGGTAGAAGATGCTA
AAAATTGCAAGAAACAACATCTTTCTTCATACATTTTTTTCGTTGGTTAACTTCCATTCTTTTTTGTTTTTCTTTATATTTCAAAATAGTCT

AATTCATATACTTCAAAACTTAATTTATGTAAAATTTTAAAAATATTCACCATGCATTAAAAAAATGTTAATGCATTTTGAATATTTGATCA
TGCAATTTAGGAAATGTTTTTACTGATCTGGAGAATTTTTGTTATTGTTTTTTAATTTATTTTCTTCCTCTTACGGTTTGTGTGTCTTAAAA
AATGTTCGTGATATTTTCAAATATGTTTATAAATTTAAAATATACGTTCATGTAATTTCAATAAAAAATGCATAATACCATTCAAAAACCGA
GCTCGTGAATGATATAAAAAACGTCACATGTCTCAAAAAAATGTTCATGAATATATGTACTAGTTTTTATATCATTAAAAAAAATGATATAA
ACATTTTTAGTATA**CACGTG**AAACATGTTTTACAAACGTTGAAAGTTTTTTAAATGCATGTTTTAAACATTGCGAATATATGTTAAACATTT
TTTAATACATGTTGAACATTTTTAACGGCGTTAAACATTTTTTTAAAGTATGCAAACATTGCTTTACGTTATATATATTGAAATGTCATAGC
ATTGTTTTTAAATATGTGAAGATTTATTAAAAATGTCATGTATATTATTTTTGATGGATATGAAATATTTTTTTGGGAATCACTGAAAGTTT
TACACTTCATGAATATTTTCAAAATGTGTGACGAGCATTATTGAAAATTTGAGTGAATTATATTTTTAGTATTTTACATATTTATATAAATA
ACATCTTTTTTTAGAAACGTTTAGATAACAAACGACATTTGCTTCGGGAGCCACCGTTTCTCTCCAGATGGGCCCTTACATTTGGGCCATTT
AGGACGTGCTTGAGGCTAGGTGTTCTTTCGTCTCAAATTTCTAAAAAAAAGCGAGGCAAAGACACGTCCAGGTGTGGAGCTTCAAAATATTC
TTCCTTCTTCCTCGAGTTACTTCGCGAGATTTCCAAGAGTACGTATGGTGCATCTTTTTTTCTGTTCGTTGTCAGTGCTACTACTTCTGGAC
GAAGAAACGTCCGATCTCATCGAATGGAGCGACGCTGCGTTGTTTATTCGTGTGGTTTCCTTTTGCGGTGAGACAAGCATCG**CACGTG**ACAA
CGCGCACGCAAAAACAAATCACGCCGAGATCGAGAGCAACACGTCGACGGATCAGAGACACCGATGCATGCACATGGCGGCGACGATGGCGT
TCGCGTCCATGCTCCACCGCACCGGCACCAAGTGTAATAAATCGACCAGGCAGAAAGCCGCATCCAACTTGTGCTCGCTCGGCACCGTGGAG
GTGCACCGTAAATTGTGCTCGTCGTCGTCACACGCATGCACCACCCCTTTACACTTGTTTATTGCAGCTTCTTCACCCGCTTTTGGCCTCTT
CTTCTTCCGGCATGGGCTCGGTCGAATCGGTTCCATCTACATGGCAGAGCTTTGCGAACGTACGGCGGGGGAGCGGCAACGCGTGTCCTCCC
TACGTGGCGGCCATGTACGAGCACCGCCGCGCAACGTGGCTCACAGCTTCTGCCTATAAAGGCCACCTGCTCCAACCTCCTTACCATATCAC
AAGAAGCCGAACCATTCCAAGTACTGTCGAGCAGAAGCCTAAAGCGAGTGACATTTCCCCCTTCCGAGCTAGTTCAAGAAACCAAA**ATG**

Germin-like protein 4-1 (NCBI accession no. XP_020167477.1; IWGSC accession no. TraesCS2B02G481500.1)

ACTAGGGTTTCACCGTGACCGTCGATCTGAATCCGACGGCCGCCCTTGCTTTTCGCCTGATCAAAAACGATCGATGCGGTTGGGGGCGAGGG
GAACCCTAGTCATTTCCTCCCCCGCCCGCCGCTCGCCTCGCTCTCGGCCGCTCGCTCCTCTCTCGCCCGCTCGCTCGGTGACGGCTCCACGA
GCCACGCTGGCGCAGCGAGCCAGGGCGGAGCGGCGAGCCCAGGCGGCACCCCGATGAGCCAGGCCCTTCCCCTCCCCTCTCCGTTTCCAAGA
GAGACAGAGAGGATCCCCGTTTTTCATTTCAGCGAGAGGAAGAGCGCGGCCAGATCGCGCCGTGGCTATCCTCCTCGCCGGTGGCGCGTCCT
CCCTCGCGGTGAGCGCGCCGCCGCCGAGGGATTTGGCCGCGGTGCTCACCTCCCTCCTTTTTTTGTGTCTTTTTCCTTTCTTTTCCGCGAGC
AATTGCTAGCTCCGGTGGTTCTTCGTCCCCGCACCTCGGCTTGCCGATGCGTGTGGGGATCGAGAGGAATAGGCGCGGCCGTGCGGCGGCGC
TCTTTGCCGGTGAGGCTCAAGGCCAAAATCCGGTGGTTTACTTTGCCCTGTCTAGGGTTCTTGGGGGAGGGGAGGTATTTTTCTTTTCTGTT
TTTGTTTCTCTGTACCGAAATCAACTAGCCCGATCTGGCTGATACCATTGATAGAACCTTGTGATCGGAGTAGGCTAGTAGATCCGGTAACC
TACCTTCTCCTGGTGCGGATGAACTCGATCCAGCACCGGCCATGGTGATGGCGGAGTGTGGGCGAGGTGGTGGCGGCGGAGCTTCCCGTCGG
CCTAGCGCAAACCCTAGATCGGATTGGTGTATCGGTGGTGTTGTGGTGCACGATCAACCTCGTAATCCGTGCCCCGGCACCCCACCTCTTTA
TATTGCGCTGGCGACAGGGGCCCACCAACCATTGTTTGGTTGGGCGCCCCCGATCAGGGCGCGAGTCAGGGGCCCGTTCGACCTGTTGGGTT
CGAACGGGATAGAGATCAACCTAACAATTACATGTCCTATGGTTTTACCCAATAATAAGGTTCGGTGGCACCTATAGATAACATCTACATGT
AGGTATAGAGCTACCTACGATTTAGTCCATATCTCAATGCATCACTACGTTTGTTCGAAGAGCAGTTTTGATTGATGAAGGAAATCAAAAGG
ACCATGACTAGCATGGTCGTCCAGTAGTCCGGACCAAAGAAACTAAGAAAGACGATCAAAGTAGAATCAACATCAGTGCATCAGCTAGCTAG
TCATCTGCAGATCCGGTGGGCAGAATGTTTTTATGATGACGTCTACCCAGTTGGAGTTTACGACCAATTTTATTTTCTTCCATCATTGATAG
CGGTGTAACATTTGAATAGGACCTCCTTCATCTTAGGCTTGCTTTGTTTTTCTATTTTCTGTAGAAAGTTGTCACTCTATTCGCTCATTGCA
GTTGTATCAACTCCATATAACAAATGAATGAAAATGTTCTTTATCGAAGAAAAAAAAAGCTAGCCGACTGCAAGTGCGCCAGCAACTAGTAG
CCGGGAATTCCGTCACCACGACCTCAAGGCGTTCAATCCCCACTATCATACGTACCGTCCTCGAATTCCAGCTTCTGCAAGTTACCAGTGGA
TCCATGCTGCAAACTAAAACCCAAAAAGGAACCATTATCTTTGTCAAGCTCTTCCACCGAAAATGGTGCATGTATGAATTTCCACAATGGAT
ATTACG**CACGTG**GTCACCCTGTATAAACAAAACAGTTAGTGGGGCTGTGAGAAATAATCAGCCGATCATACGTAAGCTAGCAATGTTAGTAG
CAGTATCGTGGATGCTTACTCAAACTACACGGTGATATTAGTGACCGATTATAATTGTTGCTAGTGTACCATGTTGTTGGTAGAGCATGCAG
CTAGCCCACTACTCCCCGGCCACCTTTATGCCTGTATAAATAGGCACCCCGATACGTGCTTCCAGCGTACAATTACCAAGCTCAACACACCA
GCTTAATTACCCTGACAGACATTGTTAGGTCTTGTGTTAGTGAAGCTAGCTAGGTAAGAAAAACC**ATG**

Prx113 (NCBI accession no. ACF08092.1; IWGSC accession no. TraesCS2B02G124800.1)

GCCAGCGCTGTTGACGGCGCCGTCCGTTGCCGTCAGACGGAAAACAACGCCGATGGCTAAACTATGCCGACGGCTCTCCCACGGCCGTCGGC
ATATGCTCCTATGTCGACGGCTATACTACGCCGACGGTCTGACACATCTACGCTGACGTGATCTACGCCGACGGGGATATGCCGATGGCAGC
CGTAGGCATAGATCTATGCCGACGGCAATGGACCTATGCCGACGGCCCTGGGCCGTAGGCATAGCCCGCGAGTCCGGTGGTGTTTGGATTTT
TGCCCCAAGGATGCCCATTCAAAAATACCAGGATCAAGAGTTATGCTACTTTAGCTTTTGCAGGACCCACATGTGATAATTGTGGTTAAAAT
GCCCAAAGAGACTGATCAGTGTTTTTTGTAAAAGATTAGGCAAAGAATCAGTGGTTCTGGCCCAAATTCATAAACTGGTGTTTTGTGCGAAC
CTTGGCAACAATATGGTGGTTTCTTTTGCAATTCACTCGAGTTGGGCAGCTATCTACGTAGTGCAAAACATTCAAGACAAATCCGTTGTTGG
ATCGTGAGCCAAGAAAAGAGCTTGACTTTTGATGGAGTCCAAGCCTTTCATATCATGGGATCCATCGGGGAAAGAGTCATCGCAAGAAATTG
TGCTTATATATGCGCTGGACGCCCAATAGATCTCATCATTAGAGTGCTTCCAGAGAATGTCATCCTCCACAAGTTCATCAAGAGGGAAATCA
TGCACAAGCATCCAAAGAGTAAAAACTCCCATATGTGGGCAGCGGAGACGATAGTGCTTTATTTGATCATGAGGATCCAAGCGTTATCTTTA
AGGGCTTCAAGCATCTTTCAATTCTTCCTTCTCAAGGCTTCAAAGATGTGGTGCGATGTCCTTAGGCTTATTCCCAACGAGCCATAGGGAGT
CCCAATATGGTGTCTTTGCGCCATGGCTCACGGTGATAGTGGTGGAAGCATATAAAAATATGGGTCCTCCTCAGTGCACGGATTTTCATGCC
CCACACACAGTTTGTTGGGCTCTTTTCATTCATACCATGGCCAATGAAGGCGCAAAGCTCTTACAAATTTGTCAGTGTTTAGGACGCCAAGG
CCACCGTACTCACTGGGGCGGCATACAATCTCCCAGCTGACATTGCGCTTGGCGCTGATCGTCTTGTTGGAGCCGGACCAAAGAAAAGCCCG
GTCAATTTTGTTGAGGTTTTGGACCGTGCTCGGCGGGACGATGAGAGGCGTGATGGAGTACACCGCTTCGGAGGACACGACCGACTTAACAA
GCAAAGTTCGACCAATGTTAGTTACAGTTTCTGGAAAAAAAAAATACAGACGGTGGATGCCGCCTCAATCTTCCGGTGTCCATCTCCCCCTAT
GCTTCAAGAAGCCAGGCTCACCTCTCAGCGTCCCAGCTGCGGAATTGTCTCGTGTTGGCCGAAGGCGACGGGAGCGCAAGATGACTGCGCAC
CGGTCAAAACGAGGGTGAATCGCCTTGGAACAACGCACACATGGCTCCAGGCGTCCCTACTATACTATTCGTACGTACGTAAGGCCAAACGG
TTGGTGTCCACGCACACATGGCTCCAGGCGTCCGTGAGCATGGAAGTGATGAAAAAAGAGAGATCGTTCAATGTTTTGCATATATCTGCGCG
ATAAACTCCAAGAGAGATCGCTCAAGTTGGCCAGGTTGGTGTTGGCGGTAGCGACTCAAGCTGGAAGCTGCCGCAGCCTAAGCCTTGGATGC
ATGCGCGACCCTTCCTTCCAAATAAGCTTGTCGATCGATGCCAATATGCCATGTACCTAGCAGATTGCGGGGTACCCGATCGAACGGCTAGC
TATATCGACGTCCTTCGAGCTACCTAGAGACTAACGTTGGACTAATGGGACGTAAATATTGGAAGCTCTATCCCTAGTGGGAGACCGTGGGC
GATCTGGATATG**CACGTG**AGCTGAATCTTTGGCTATAAATATGCATGCGATACACTACTCTCTTTCACAAGCCACCAAGTCACCACCACTCC
AATAAGAAGTGCAGGTAGCTAACC**ATG**

Glutathione S-transferase 1 (NCBI accession no. P30110.1; IWGSC accession no. TraesCS3B02G152300.1)

ACCGGCGATCTGCTCCTTCTGGGTGGGGGTCGCCGGCTGACGTTCTGTTGCGGGGTGGGGGTCGCCGGCTGGCGTTCTGCTGCGGGGTGGGA
GTCGCCGACCGGCGTGCTGCTGCTAGGACAATCGGTGAGGCCAGTTAGGTGCTAGCCGATCGATTGGCGAAGAGATCCGAGTCCTGGGGAGA
TCAGTGAGGCCAGGTGCTATTTGGCCTATCAATTGGCCAGGTTCTGGGAACGGGGCGTGGCGTGATCAACGAGGTGCTAGGCTGCTAGCTAG
GGAACTGGATCCTGGAACGTGGAGGAGGCAAGTCCGGTATGCTAAGTACTTTAACTTTCCTTCTTCACATCCACCTGATTCAGATTATTTTG
ATCTAAATTAACTTGCAAAAAATATATGTGTGATATCCATCTACTATAATTGCTTACAATCAAAATTATATGTGATTTTTTTTAGTTTAGAA
GATTTATATGCACAGTAAATCTGAATGTTCTTCACATGCATGATTTAGTTTAACTTTAAAGAGTTATACTAACTAGTCTTGATAAAGAGATC
TTTTGGAGCAACACCAAACCTCGTGAGGTGTTTTGCCTACGGAAAGGTTGTGCTATGTAATGATTATTATTAGGATCAAAGTTGTAGGATAA
ACGTAAAACCTTCTCGATGTATCTTTTATACAACATTGTAGTTTAGTTATATATGGAGAGAGTGATTTAACACTTTGTGTTTAAGAGTAGAA
TAAGTTATTCCACACTCTAGCCAAACGAACTATTTGGCAAATATCTCGCTAGCTGGTGAGAGCCAGAGCCGTGGAAAGTCTGTCTTGCTATT
AAGGCACAAGCATCAAACAGGAACATTTAGAGCCATGGAAAAGTGATGTGTCGCCTACCAATGGGCCAACTGCTAGCGATGTAATAATAGCA
TCCAAGTTGATTTTTTATAGAACATGCAAGGCGTTGGCAAGTGGGAAAATGATTGATCGCTGGCAAGCTTAACTCTCGGAACTTATAGCATT
CAACTGAATCAGAACAAAGATTTAAAAAAAAATACATTTCCATCGATAGTGAAAAATTATTCAATTGAGTGACAACGAAAATCATATTGGAAT
GTACATTTACTTGTTGATTTTAAATTAGAGGCATTTTTCTACCTTTTTTAGTTAATAAGATATGCATATACCCACCCTTAGTGTTTTCGAGA
CAACGAGAGGGCACATTGCTTTTGGTGCTACCATCTCTCTCAAGCCTCAAATAAGTTGTGCGGACACGATTATCTTCCCGCGTTGGAATATC
GTGGCCTGGTAGAGCTAGCGAAAAATCTTCCATGTTGGAATATGTCGGCAGCCGGATAGCCGCCATGCATGTAAAGTCTCTTTTACCTTTAC
ACTTGCTCAAGTGACACTGTATGTCGCCTACCACTTGCTAAATCAATGGGCCAACTGCTAGCGACGTAATAGTAGCAAGTTGATTTACAGTG
TTTTGCTACAGTTCTCTGACTTTGTTTCTTCATTTTAGACTAGCTGACTACTGTCGCTTACCTGCCTTCCCTTCTCCACGTTAGAGGATCCA
GTTCTGATATTGAGACCTCGACGATGGGAGGAAGGGCGCGATCGATGTGGAGTAATTTGAATTTCAAATCTATCTATCTGGGGTATATTGGT
CCTTCACCGATGTTTGGGGGGCTGTCGGAAATTGGTTCCGCGATCTACAAAAGTGAATGGAGGGAGTAGTTGTTTCTCCAATCCGTACCAAC
G**CACGTG**TTTCTAACTAGTACTTACTTCCTTCGCACCACAATATGGAATAGAGGGAGTATCGATAAACTAACAAAGATGATTACTTACCCGG
TTTAAATGATTCAAGAGCTCATTTAATTTGGCACTCATCATTTCATATATCTTTTTTGGTAGAAATGAAATAAAGCAGATCTAGACACTAGC
TAAAAAGTCGATGTAGCCTTGTTATTTCCTTGGGCCACGCGGGCCGGGTGTGGTGCTCCCTGCTCTGTGTATAAATGGAGATCAACATCCAA
GGCCTCCTCCCACACACACACGCTACAGAGCAGAGCAGAGTCTTGCTCCAGTATCTGCCCTCTCCTGCCTGCCTGTAGAGCATCCAT**CACGT**
GAAGTTCACGGACAAAC**ATG**

Peroxidase (NCBI accession no. CAA39486.1; IWGSC accession no. TraesCS2B02G125200.1)

CAACCGATGGGTACTGCTCAAATTTGCCCATGAAATCACGAGCAGTATAGGTTGCACTGCTTGGCTCTCGTCGTCTAGTATACATGTCATAA
GGGTATTTTGGGTAGAAAAAGTTTATGCCGGTCCGCCAGGCGAAGGCCATCTATGGCGGATCAAGAGGCTGCAGAGCTAGGCCTCTCTTCCT
CGCCATCTATGTTACGTTGTACTCTCCAGTCTCTCCCTCCCTCCCTCCTCCTCTTCCTCCCACCACGAGGAGCCCGAGGAGGACTCGCCGCC
CAGGACGCCACTACTCCCCAGGGACCTGACGTGTTTCAGGACCGGTCGGCACTCACTCGTGACTGCTCTTGCCTCGTCTTCGTCTCCACGGA
GGTCCCTGCCAGCGTCCCGCCGGAGCCGTGCACCCGTCTACTCCGGCGACCTCCCCTCCGGAAGCGCGTGCAGCCTCAACCCGACTGGTCAA
ATGAAGCTGCAGGACCTCCGAACCCACGTCCAGGAATTCTTGACATCCCATGGCTTCCAAGTTCCAACGAACGCCTGCTCATCCGCTACAGC
CGCCCGGTTGGGCTGGGCAGACGAGACCATGGCTCCCTTAATTTTTAAACGAAGAATGTCTATCCTACCTCAGAAATTCAATGGTCAGATTT
TAATATACTGCTCCCTCCCCTGTCAGCAGTATAGGGTACTGTAAAAACGCACTTAGAAGAAGCTTAGCTCTCACCTTAGAGTGGTCCAAATG
ACCCCTCTGCTCGATCATTGCACTTGATGTTATCGCTATGCCTAGCCTTGATTGATCCATGCGTGGTCACTTGCTGTTAGAGATCAGATAGC
TACTTCAAGGTCGTGAGTTTATCCTTCGGAAGATTAGTCGTGAATAGAACAGGGTTTATGGTAGATCCCATGGTTGTGCCTCATGTTGGTTG
CACCATACACGGCTCAGGGTAGCCGATTGTAACCCTATTATATAGAGGAATAAAACTCGCAACTTTCTTGAAAAATAAGTCTGTTTCAGGAA
TCCAAATCCAACCAGCAATTCGTCGGCTTTGTCGAAAAAAAAACCAATAATTCGTCGGAGGCATGGAAGCCAAAGAAACTGCGTCCAGCCTA
CAGATCGGGCCACGTTACGGCCGGCCTAAACGCCCGTCGGTGCTGGCCTACGGCTGTGCGGCCTCGGTCCAGCCTCACTATTCCCACCGTGC
GTTTGATAAGGCTCAACCGCTCAAGGCAGCCTTACTACCTCCGTTCTTAAAAAAGTATACTTTCAACTTCATTGAAGGGTCAAACTACTCAA
AGTTTGACCGAGTTTGTGCAAAAGTATATCAATGTTTACAAAACCAAATAGGTGTATGATAAAAATATATTATATCATGAATTTAATACTAC
TAATTTGATGTCATAAATGTTTGTATATTACTATATAAATGCAGTCAAACATAAAAAATTTGAATTTTCAACAAAGTTGAAGGCACACTCTT
TCAAAAACGAATGGAGTACCTCTCAATGTCCCAGCTACCGAATTGTCCGTGTTGGCGGAAGGCGACGGGAGCGCAAGATGGCTGCGCACCGG
TCAAAACGAGCGTGAATCGCCTTGGAACAACGCCGACTCCCCACTACTATTCGCACGTAAGGCCAAACGGTTGATGTTCACGCACACATGGC
TCCAGGCGTTCGCTAGCATGGAAGTGGTGAAAAAAGAGAGATCGTTCAATGTTTTGCCGATTTTTGCGTGATAAACGCCAAGAGAGATCGCT
CAAGTTGGCCCTGTTGCAAGGAAAATTCAGGTGTTGGTGTTGGCGGTAGCGGCTCAAGCTGGAAGCTGCCACGAATACGCCAAGCTGGAAGC
TCGAAGTATACCCAATCGAACGCGAGTCAGAGACTAACGTTGGACTAATGGGACCTAAATTTTGGATGAGCTATCGCTAGTCGGAGACTGGA
GCGATCTGAAGGTG**CACGTG**AGCTAAATCTTTGGCTATAAATATGCATGCGATGCACCACTCTCTTGCACAAGCCACTAAGCCACCACCACA
CCACTCCACCAGTAAGAAGTGCAGCAGGTAGCTAGTAAGCCGGCGTAGCTTTGCTCTTGCAGCTAGCTAGCTAACC**ATG**

Probable xyloglucan endotransglucosylase (NCBI accession no. XP_020152513.1; IWGSC accession no. TraesCS7A02G426700.1)

ATGATCAACATAATAAAATTTCCATGCTACCTATAAAAAAATTACAGATGGAAAATTTAATAAGCTTGTTATTTGAAAAATATGGAATATTT
CAGAAAAAAATCTCTAAAACAATCAAGGCAACCTTTGGAATTTTGTAATTCGCACATGGCAAAGTTAGAGATTTTTTTTGTTTGGAAAGATG
GCAAACTTTAAGAATTTATTTAAAAAATAGCATGTTATGGGCTGTATATGTGTTGGATGCTACGAGGAAAAAAATTGTTCGTGCTTCACATC
TTTGTTAAAGATGGTGTCATATTAAGTTCAATTTAAATTTTTTCTCTATGTGCACATAATTTTGGACCATTGTTTTTGCATCAATTTTAGTT
GTTTTGAAACACCTACTCCCTCAGTTCCTAAATATTTGTCTTTTTAAAGATTTCAAATGGATTACCACATAAGGATGTATATAGACATATTT
TAGAGTGTAGATTCACTCATTTTGCTCCATATGTAGTCACTTGTTGAAATCTCTAGAAAGTCAATATTTAAGAACGGAGGAAGTATTTATTA
CACAGTTGTCACAACACTTGGGGCATCATCTAGTTTACTAAACATGGATATACATAGATCCCTATCTAGTAATGGAATAATTATTCCTAATA
TTTTTAAGGATTATGAAATACTTATCTTA**CACGTG**GTCTACATGTATTGTTCTTCACATTTTTTGTAGTAAGCAAACTGATTTTTGTGATTT
TTCCTTACATAAAGAAGCACAATCGAGTAAGCCAAATAAAAAATAAAAATAAAAGTTACTTCGGTATCAAACTATCAGAGTCAATGCCTTCG
TACCTTTGAAGTGAAAATAAATTTCCTGGTTTCAAATGGATGCATGAGCAATCAGTTCCATTATTCCAATAGGTGAGAACCTAAATTAATTG
AACACGTCCTGACCAGAGACGTGGCCCTTGCTAGACTTGATGTCAAGCAATTTTCTCATCGTTTAGATTGTTGAGGAGAAAAATCAAGGCTG
CTAAAACTAGATGATACCTCATGCAGTGTTACATAAATCGGTTGCCTTACAATTCAATGAGATTTGTTTGTACGAAACATAAATATTTTGAT
AAAAAATGAAAAGTGGAATTGAATATACATATTATTTATTGTATTTATTTCTTGTATATAGTTGTAAAAGATTTATTGAATGTAAGTACCAT

AAAAAGCATTTTGTCATACATGATTGTATGTGACCCTTTTTCCTATGCATGATTACATGTCGAGGTGACATACTTCCATTGTAAGATAAATA
GGTTAATGAAAATAACCTACTTATAATTGTATAGCATTAGAGGATTAGTGACCAGAACAGCCTTAACATGAAAGTACTCCCTCCGTCTCAAA
TTGCGTATCTTAAATTTATCTAAATACGGATGCATCTAACACGTAATCACTAGCTAAGGGCATCGATTGCCAATCCCAAATAATAGCAACGA
TAGCTCTGAGACATGAGAGAGTTCTATCCATTTTGTGTTGTCTCAACCTATCTAACATACCAGGAATCATCACACGCTTCGAGATGAAAAAG
ATTGTCGCAAATAATAGGTCGTCCATCCATCCCTGACTAATTCTTGTTCATGCATCATGTATGTTTTGTACTGCCATTTGGGTACAAAACAC
AAAATCATCTCAAATAATCATATAATAAAATCAAAAGCTGTTGCCTTGCCTCCTGAAATCTGCTATACGTCTCGATCGATGCTTAACGTCGT
CTTGCTGTAGGTGAGAGCCTCTGGCGTGACCATGTCGGGGCAGTCAGGCGTCTCATTTTCCATCAGAAAATTGTTCGCTACTCACTCGCCAC
AGTACCGTCCTGCCCTGTCTCCTCTCCTCTGGCGCCAACTCACACGCAAATACACCCGAGCTGGCGAACTCCTCTCCGATTCGCCGAACCAT
CCATCCATCCGGCACGCCACGCTTATCTCCAAACCACTATATAAACGCCCTCACGTCCTGCTCATTCCACTCACACAACACAAGCTTCCATC
AAGCTTTCTCCTCATCTTCATCTTCTAGCACTTGTTCTTCCTAGCTAGCTCTAGTTGGGTGAGCTTACTTTTGGGAGCATCGAGCCCGCAAC
GC**ATG**

Serine/threonine-protein kinase CTR1 (NCBI accession no. EMS46272.1; IWGSC accession no. TraesCS6A02G344800.4)

AATGTAGTGTCGTGATTAAAGAAGATATCTCCATAGATATGGACGGCACAGAACGTAGGTAGGACAGAATGAGACGAGGATAGAACACATTT
GTGTCTGGCCGATAATCTATTGTTTCGTTGAGCGTTCTTTTCTGCAGTGGCAGCAATGCCTTTTTTCTCCTTTTTGTTCGAGAGATCAAAAA
ATAGCAGCATCGATGCGCCATGCCGCCGTCGATCCGATCATGCATGCAAGAATATATTCTGTTCGGCCATGCATGTCCGGCTTCCAGAAACT
CTCGTGGGTCGTGGCCACTATGTACAATATTCTCCCATGGATCCACCGGAGCGTATATCATCGGTTGATGCCGCCCTCGCTCTCCAGCATAT
GCAATGTCTGGAGTGGAGCGTGGAGCATCTGCAGTATGCGCGTACGTACATGTGTGAAAGTTACGGCCGATCTGATCTGCTAGCTATACCTC
CTAGCCCAGGAGTCGATGGTCAGTGGGTGGGTCGTGCGCTGAACTTTCGTGACGTGAATGCCGAAAACGACGGTCGGTCCGGGCCGGCGGTC
TCGCTCCTTCATGTTGCCTGCCCGCCCCCTCTCCATCTCCGGCCAGCTGCCGTGACCGCGAGAGTGAAAAACGATGGCGCGGAAGCACGCAGG
ATGGTAGTATAAAACCGGGAGGGGAGGGGCGGGGGTCGCGTCATCGGGAACGGAACAGAATCACGCCA**CACGTG**CCGCCACCGCGAATCCCG
AGCCGCTCCAATCCCCCGAGGCCCAGCCCGACCCGAGTGGCCCGGCGCCGCGCCTCTCTCCCCCGTCCGTCCGCGCAAACAAACCACATTCC
CTTCCCTCCCTCCCGTCCCACCACCACACGTAGCGCGCCGCACCCGCACGGGCGGCCCGCCTCCACACGACACCCAGGCAGCCGATCCCCTT
CCACACCTGACGCCGACGCCGACGAGTCCCGCGCGGGTGGCGGCGCCGGCGATGGCCCCCGCGCTGACGGCGTGATACCTAGAGCCAGCCAG
CGAATATCCGCGCGCCCCGAATCCCCCAACCCAGCCAACAACACCACGGCCTCCCCCGTCCCCCCCCTCACTTCCCCACCGCAAATCCGGAG
GACGCATCCTCCCCTCTCACCCTCTCTTCCCCCCCTCGTCGCCTTTCCCGCCCCTAAGCGCAACCGCCCCGCCGCCCGGAAACCAGCCGCGC
TAGTCGGCTGCTCGCCCCCTCTCCCGCTTGCTCAGCCGACCNNNNNNNNNNNNNNNNNNNNNNNNNNNNNNNNNNNNNNNNNNNNNNNNNNN
NNNNNNNNNNNNNNNNNNNNNNNNNNNNNNNNNNNNNNNNNNNNNNNNNNNNNNNNNNNNNNNNNNNNNNNNNNNNNNNNNNNNNNNNNNNNN
NNNNNNNNNNNNNNNNNNNNNNNNNNNNNNNNNNNNNNNNNNNNNNNNNNNNNNNNNNNNNNNNNNNNNNNNNNNNNNNNNNNNNNNNNNNNN
NNNNNNNNNNNNNNNNNNNNNNNNNNNNNNNNNNNNNNNNNNNNNNNNNNNNNNNNNNNNNNNNNNNNNNNNNNNNNNNNNNNNNNNNNNNNN
NNNNNNNNNNNNNNNNNNNNNNNNNNNNNNNNNNNNNNNNNNNNNNNNNNNNNNNNNNNNNNNNNNNNNNNNNNNNNNNNNNNNNNNNNNNNN
NNNNNNNNNNNNNNNNNNNNNNNNNNNNNNNNNNNNNNNNNNNNNNNNNNNNNNNNNNNNNNNNNNNNNNNNNNNNNNNNNNNNNNNNNNNNN
NNNNNNNNNNNNNNNNNGAACACGAGCACGCACGAACAATTGGCTGGTTTTCTCCTGTTTTTTTTTTGGCAAGTTTGTTAGTTCGTCCCTCC
TTATCCTCCCCGTCTGTTTCGGGGGGAGGAAACGGAGCGATTTTCTGTTTTTATCTGGCCAAGATCGGCGGGGAATTTTCCGCCCGTGGGGA
ATTACAGGACCTCAGTCCCTGTAGATTCTGTTTCAGATTTCGAATTCCGATTTTGCGGGCAAATCATTCAGGTCTGCTCCAACGCATTCTTC
GAAATTGCCAGATTTCGAAAGGTCGGCTCATTCTTTTTTTAGGAAAGAATTCTGTCTCTTCTCTGACGGTGGGGGGCTGATCTGTGGTGTTA
TTAGGGAAATCTATGGCAAATTGTCCGATTTCGTGTCCGGATTTCATGTCTTCATTTGTCTGATGATGGAAGCTGCCAAATCTTTGGTCTGT
AGACTAACAATTTCGGTATTTTTTCTATCGACAGGAAGGGGCCAAAACACGAGCAGCTGCTTGGGACTGGTGGATTGGCAATCGTGTGATGT
ACTTGGCTCTTGCGCCGATCCAACTCAAGCCAGCTTGATTACTCACTCTCGGCACAC**ATG**

Putative acyl-activating enzyme 19 isoform X1 (NCBI accession no. XP_020199323.1; IWGSC accession no. TraesCS7D02G030200.3)

ATACCGAATACCGACAGCGCACTAAAGACAATCACGAGGCAAATGCCCTTTCCGAGACCTCATTGCAGTAAATGTTCGAATAATATCCTTAT
CATCAACGGACTTGAGTACCACCTGCTCCTTGTAACTTGTAACACATGACTTGAACCATTCATTATTGATCTTGCTACGCAGTTTGCTCCTT
GTTAAAAATAAGAAAACAGTTTTCTCTCTATTAGTGCTCGACAGAAAAACAGAAGTGTTCATAAAACTTTAGAAAATGACAAATAAAAAATA
CAGTCATGCAAACTTAAAGAAAATGACATGTTTGGCTTGAAAGAACAAGGATTATAGGATCAGGAAAATCAGCTTTATTGACAAAATCTGAT
CTATATAACTTTAAAGTAGATACTTGGTTGCAGCTAAAATAGAATCAAGTGCTGCCGGCAGGTCCGGTCCTCGGATTTTAGGGGCCCAGGGC
GAAACAAAAATCCAGGGCCTTAAGTACAAAGGTCTAAATAATAATCAATCTATGTAGACATAAATTTAGAGGCCAAAAAATAAGGGCATCTA
AAATCATTAAGCATATCAAATAATAAAATCATCCGAGATGCAAATTAGTAAATTAGTAATGATAGTAACTATCTATCTCATCCAACAATTTC
TTCTCAATGCATACTTTCGAGATGGCATGACAGTACTAAAATTGAAAAGGACAATTATTTTTGGAAATTAGCACAAAGCTATGATAATATAA
GATCCAGTTAAAAATCATCATCAAGAATCTGCCTCTCGCCTCTGGTTCATAAAACCTGCTAGGGCATACCTTATCTGGAATCTGCCTCTCAC
CTCTTCTTGTGGTGAACGGCGTTTATTATCACCTGGACGGCAGTGCTAACGACCTGATTGGTAAACTATAGAGATTAGAGAGGAGAAACAAT
CAGGCAAATCGTTGGACAAATCTAGAATCAGAAACGCATAGGTCGCTGCTCGTTCGGAGGAAGAAAGATGAACAGCCAGATGTATGTACCTG
GTGGCGCTGACGTCCTAGACTCCAGTCGAAATCAAATTAGGGTGCGGCGGTGCGGACACACCAGGAACCTAATTAGGGTGCGACGAGGACGA
CACGCTGGATCCTCATAGGGAAGGCTCCCCCAAACCATGGAAGCTGGCGTGGTCGCGCGGAGGACGATGATGACGACGGGAGGAAGCTGAAG
TGGGTGAGGTGGAGCTCCTCCGTACGCGCACATGCCAACACCCGCCACCGCCTGCGATGAGGACGACGCGCCGGCTCCTCATAGGGAATCCC
CCAAATCGTGGAATCTGGCGGGGTCACACGGAGGATGATGATGACGACGGGAGGAATTGGGGGAGGTGTAGCTTCACCATGGCCGAGTCGAG
CGAAGGAGGCCGGCGGTGGCGATTTTTGTGGAGAGAAAGATCCGATCGAGATCGAGGGGGGAAGTGGAGGTGCAGGCGTGCGTGCGAC**CACG**
TGGAGACGCGAAATCTACCCTGTGCCGCAGCGACGGCGTCCCCGGAGTTCGGCGGCAGGGATTGAAACCGCCCTGCCCTGGAGACTTGGCGG
CGGCGTTTCTCGGAGTTCGGCGGCGGGGGCGGCGTCTCCCTCTCGGACGGCGGTGGCGGGGGGCGGCGTCTCCCTCTCGGACGGCGGCGGCGG
CGGCAACGATAGATGGGATCGATGGAGTTTTTTTTTTCTGGGTGTGGGGTTTTTTTGTGGCTGCGTGGATGGGTGGGGTGGGTAGAGGGTGA
AAAAAACCAGCGAAAAAAACCGGACGAAAATGGTAGGACGAAA**ATG**

Aspartyl protease AED3-like (NCBI accession no. XP_020167595.1; IWGSC accession TraesCS2D02G132500.1)

AAGAGGACGAGTGCGACATCGAAGTGGAGCCTTTGTTCGAGGACGAGCTCGCCAACCAAACCGCCTGTCCTAAGCCGAAGCGCAAGAGCAAG

CGCACGAAGGCGTACACAGCTGCCGAGGACAAGCTTCTTTGCGAGTGTTGCGAGACATTGGACAAGACCCAAAGACGGGCGCCGAGCAAAAG
CATTCGACCTTTTGGACTCGTGTGCACCGCGAGTTTCATGAGCGCAAGAAGTTTCCACCTTACCAATTTGTGAGCACGCGCGGGTGGGTCTC
CATTTCCAAGCGGTGGAGGGTGATCCAACAAGAGTGCAACAAGTTTTGTGCCACCCTTGAGAGCGTCAAGGCCCGTCCCGTGAGCGGCATCG
ACGTGCAAGACATGGTATGATAGCAAGCAAGCCGCCCTCTTTTGTGCCATCAAGATCATTCTTTTACGTCTTTATTTGCTATCACTTGCCCA
TATGCTTGCATGGAAACATTTTGTAGGCATTTCAAGCTTTGGAGGCATTCAAGGTTCAACACAATGGCAAGTGCTTCAACCTCTCCCATTGC
TATCGGGTCATCAAGGACGAAGAGAAGTTCAAGACACAATATGCCGCGCTTAAGG**CACGTG**GGGGAAGGGCGCCGTGGAGGATGTTGGGGAG
GGCGAGTCGGCACGGCCGCGGGGGAAGACCAACTCCAAGAAGGAGGACAAGCGAGATGCGACCACCAACACCTTGATCGCAAGCGTGGACGG
CATGATGAATAAGAAGGACTCAAGGGAGGCGGAGCGCCGGCGTTTCAAGGCGGAGCAAATGGATGCTTTCATGGAGATCCAAAGGAGGAGGC
TTGATCTTGACGCCGAGAAGCAAGCCAAGATGCTAGAGATCGAGGCCGCCAACGCCAAGACAAAGACGAAAGAAGTGGCTCTTGCAAGCATG
ATGACCGGGGTGGAGATCATGAAGGTGAATCTCAACGCCTTGTCACCAAGGAAGAGGCCGTGGTTCGAGAAGATGTAGGCCGACATGCTCAA
GTTCGATGACGAGTGATCGATGGCGTCGAGGGCCATCTTTTTGTATGCCGGTAGGTGTGCCGGCATGACCACGGGAGCCGCGATGGCGTGGT
CGAACTCAAGTCCCACTATTTTTTGTGTGCTGGCATGTGTGCCGGCCGGCTGGCGAGTGCGCCAGCATGAACTGTGGTATTTTCTGAAGCCG
GCATTGTATGCCGGCGCTGGCATGATGCCTGTATGAGACATGGCCGCTGGCATGATCGACAGCGGGCTTTTTTTATTTAAAAATTGAATACG
GACATGAAATGAGTCGGCGCATTGGGCGCATTGCCGATTCAAATACAAAACTGAGCGGACGCCGGACGTGCGGCCGACTCAAACGGACAAAG
AAAATACAAATGCGTCGTTCGTTTGAGTCGCCCATTGGAGTTGCTCTTACCCGCGCAATCCCATTCTTATTCGATCGGCATCTCCTGCGTAC
GGAAGAGGGCAT**CACGTG**TGCCGGTGTGCGTGCGCAGCCTCCAAGGTGCAAACCGTCTCCTTCGTAAGCAAGCGGCACGTACACCGGGTCCA
AGAGCTCATGGGCTCATGGCGACGCGCACCGACACCCACTGGCCAGCGCCATCGTCTCCATCGGATATTCGAGGCTCCCGGTCCATGTCCAT
GGGGCCGTACGTCGCCCCCGTGGGCACCGCCATGCAGAGACAAGCCTCCCCTGCGACGAGCAGGCCAAGACACAGGGCTCGTCGGCAGTGGG
CGCGCACGGAATTCAACGCACCTGGCGTGCGTGGGCCGCGTCCATTGGCCGGGGCCGCAGACTGAGATCACGAGCTGCCGCAGGCGCACGCT
GCAGTGCAGTGAGTAGTGGTATCGTGCTGGGTCCGGTCGCGAGTGAGAGCATGGCCACCCGCGAATGCGTCCAGTGCGAATCCTCACGGGCG
CGCCATTGGCCACGGCACGGGCGCTGGCCGTGTATGTAGTGTAGCTTCCCGCGCGTCACGGCT**CACGTG**CCCCGCTGCCCGCTCGCGCTATA
TATCCACCGTGTCGACGCTGCGCGTTCAGCACCTCACCGCCACGCTTCAAGGCTAGCTAGCTCGATCGGTACGGTGAAA**ATG**

Non-specific lipid-transfer protein 4.1-like (NCBI accession no. XP_020197700.1; IWGSC accession TraesCSU02G057300.1)

TCGTTCGATCAAGACAACTAGCCTCTGTCGACGCTGGCTGGCCATCTTATGTCGCCCCTGTTAAAAAATCGTGCTTGTTAAGACTTTCTGAA
TCTCAGAATCCGATGTGTTTTACACTGGCACGTTCTTTCTTTCCAGGGTCACTGATTCGTGTGCCCTTCTGTAATGTAGAATCCCTAAAAAA
ATTCAGGTCATATAGTCGAGGCTCTGCAAATAAGCACAAAGACACATCCGACTAAAATGGCTCACACTATGCATAGTCAACCGTTGCTCTCG
TGTTCGACCCCCACGGGCGGCACTGTATTCCACCCTTAATTTTGCAAATAAACCAGACACTGACATGTGGGTCCTGGGATGCCCGACCATCA
TTATATTCCACTATTTTTCCCTAATAAATATGCTGACAGGTGGCTCCCGGGTGCTCCATGCATCACTATATGCGCCAGGTAACGGTCAACTA
TGTTGACCGGACAGAAACGCTCGGTTCGGCATTTTTGAGTGTCGGGCGAGGTGGAGAAAAGTGAGGGATGTTAGTTGTGTGGGGCAAAACTG
AGGAGTACTAAGCAACCAAGGTCATAGGAATAGAAAATAGAAATCCCTTTTTTATTCATTACCTTTTTGTTTCCAAATTGTGCTATTTTACA
TCCCATGCGTGCACCCTCTGGACCTCTTTCCATGGTGGCATCGTATAATAGATTGAGATCCTACATCAGTGGCACATAATGAGATGAGTCTT
CTGTGTTACACCTCTCAAATTCCATCCATATTGGCATGAGTTTTAGTATATATATACACAAAAAATCAACACATATATACCACATTTTAGTA
TTTCAGCACATATATAC**CACGTG**CGATGGAATAAAAAATGGTATTATTTTTTAGAAGTAGATAACAATAAGCTTGAACTAGATGGCACCAAA
CAATTTCCAATTACAATCATGGATGAGTAGCCTCTAAGTGAACTTCTTAGAGAAACATTGACATATTTTCTTTCTTCTTTTTTCAATTACAA
GACAAACAAGAGGGACACATTTTTCTCTAATTTTCTTTCATTCAAGCTACTTGTGCAATTCCTACATAGAAATGATCAAGTGAAGGCGACTA
TATAATAAGATCACCAAAATCAAAGACAAAATTATGAGTTCACAAAACAAGATCAACACATTTAAACAACATCATG**CACGTG**GTTAAGGGGA
GAGGATTTTGGGTCGAGGTTGGCGGTTGTCTTCCCGTATGCGGATATGCCGGTACTCAGGTATGGGTATGGCCTTCCCATCCCGTACCCGCC
TTACCCACTCGGTACAATTTCTTCCCATTTATATACCCATGGGTATATATTTTCTCAAACCCTTACCCTAATAGGGTTTTTACCCGTCGGGT
TTGCGGGTTGCGGGCACCCATTGCCATGTTGAGGCATGACACATGTGGTTCGAGAAACCATAGGCCTTATGGTAGTGGCACAATCCACCCGT
ATTCGCCTCACCACTGAGCTATCAGGATCTGTTCGCCAAGGCCGTTTGCCAAATATAGTTGATGTTTTACTTAGTCAAATTGACATATATAA
TACGTTGCTATTTACTTGAAAGACACGCCCCTCCGTCCCATAATATAAAATCGTTTTTCAAGCTATGTTAGCTTGAGAAACGATCTTATATT
ATGGGACAAGAGAGTATTTCATAGAAACTAAATCGATATTCAAAATATACGTTTGAAGACGACATCGATGAGTATCAAACCGTGACATTGGC
ACATTGTCGAATTTAACATTTGAGACAGAATGAGTAAAGTCATTTAACTAGAGCCCTATAAGATCACATGCACCGTCAGATTTATGTAGTTC
ATGGTTCGAGCTCACTGTCATCAATGCACTCAACTACACTACTCCACGAGAGACTGAGATTAGGTAAACCTCTTAGTCCCGTCATTAATCTG
CTGTGCACACGCTAGGTCCATCCATGGGGCCCAACTCAACATCCAGCAAAATGCATTAACGACCATCG**ATG**

Non-specific lipid-transfer protein 3-like isoform X1 (NCBI accession no. XP_020151640.1; IWGSC accession TraesCS2A02G477700.1)

TATATAAAGGATGGAGGGAAGAGGTGGCCGGCCCTCATAGTGCGCGCCCAAAGTGTGGAGTCCTACTAGGACTCCCTACTCCTAGTAGGATT
CCACCTCCCACATGAAATAGGAAAAAGGAAAGGGAAAAGGAGAAGGAAGGAAGCCTAGTCCAATTCGGACCAGTCCATGGGGAAGGGACATG
GCCACCCTTGAGGCCTTTCTCTCCTTTCCCGTATGTCCCATTAAGGCCCAATACGAATTCCCGTAACTCTTCGGTACTCCGAAAAATACCCG
AATCACTCAGAACCTTTCCGATGTCCGAATATAGCCTTCCAATATATCGATCTTTATGTCTCGACCATTTCGAGACTCCTCGTCATGTCCCC
GATCCCATCCGGGACTCTGAACAAACTTCGTTCATCAAATCACATAACTCCTAATACAAATCGTCACAGAACGTTAAGCGTGCGGACCCTAC
GGGTTCGAGAACTATGTAGACATGACCGAGACACATCTCCGGTCAATAACCAATAGCGGAACATGGATGCTCATATTGGCTTCCCACATATT
TTATGAAGATCTTTATCTGTCAAACCGCATAACAACATATGTTGTTCCCTTTGTCATCGGTATGTTACTTGCCCGAGATTGACAGTCAGTAT
CTCAATACCTAGTTCAATCTCGTTACCGGAAAGTCTCTTTACTCGTTCCCTAATGCAACATCCCGTAACTAACTCATTAGTCACATTGCTTG
CAAGGCTTATAGTGATGTGCATTACCGAGAGGGCCCAGAGATACCGCTCCAATATACGGAGTGACAAATCCTAATCTTGATCTATGCCAACT
CAACAAACACCATCGGAGACACCTGTAGAGCATCTTTATAATCACCCAGTTATGTTGTGATGTTTGATAGCACACAAGGTGTTCCTCCGGTA
TTCGGGAGTTGCATAATCTCATAGTCAGAGGAACATGTATAAGTTATGAAGAAAGCAGTAGCAATAAAACTGAACGATCATTATGCTAAGCT
AACGGATGGGTCTTATCCATCACATCATTCTCTAATGATATGATCCCGTTCATCAAATGACAACACATGTCCATGGCTAGAAAACTTAACCA
TCTTTGATTAACGAGCTAGTCAAGTAGAGGCATACTAGGGACACTCCATTTGTCTATGTATTCACACATGTACTAAGTTACCGGTTAATACA
ATTCTAGCATGAATAATAAACATTTATCATGATATAAGGAAATATAAATAACAACTTTATTATTGCCTTTAGGGCATATTTCCTTCAAGGCA
TCACAACAACTATACATCAGTGCCCGAC**CACGTG**AGTCGATCACTTCGGTTTGTAAGCAACATATATCAAAACTAGTGTGTAACACTCTGAA
ATTTTAACCACTTATTTAAGTGAGAAGATAACAACACTATACAAAGTTACAATATTTCCAGGCACAATTTATGATTGATAATATAATTAAAA
GAAAACAGAAATTAACTACGCAGGAGTATATGCTTGATATACACTCCACTTGGTTGGAGTTAAGAAAGCACCGCACATGTTGCTGGCATTGA

TCACCGCACCGCCTGATCAGTACATATGCCGGTGGAAATTAATACTTGACTAGTCATAGATCCTAACTTTGCTTTCAACGATGCATGGCATG
GTAGATGCATAGATCGGTCGTACTCAAAATTTACTCCATATCGTGTCCATGTCCGTCCCTCTTTTGTGTTTGACAACGACGGCCTACCTACT
CACCACTGGCCATTCTTCCAGATCTGCACACTACACTGTGGACTGTGGTAGCTCGCACACAGGTTCCATACGTTCACACGCGAAGAAATTCA
CGTCGGAATTATTTGCAGAAGCTTCTGCTCATACTAATGTAGTAGATGTGAGCACTTTTCGAAAGTAAAAATGTAGATGTGAACAAAGAAGC
GTCACATGCGTGCAGTGCACCGATCGATCCATGATCCATCGTACGGTCCCCAGCGCCCAACCACCGGC**ATG**

Type 1 non specific lipid transfer protein precursor (NCBI accession no. CAH69190.1; IWGSC accession TraesCS2B02G501000.1)

GTTAGAAAAATCAGGGATTAATAGAATTAATTAAATCATAACAGTATGCGTGCTGCTAGCATACAAGTTAGCAGCGTCTGTGTTTACTCAAA
CACTGCTAGTGACAAGATTATATTAAATTGCCTTTATATAGCAGAACAACCTCATATAAGTTACACGATTGGAGATGTAACTGCAAACTGTG
TACATTACATGCATGTATCCAAATTTATGTCACAAAAATAACAACATTAACATGTCTCATTCAACACCGGAAAACTTTTATGTAACACTTTG
AAATTTTAACCTCTTATTCAAGTGGGCACATAATGATACCATACAAAGTTTCAATTTTTCTAAACATCGTTTAATTTTGTAAAATTCAAAAA
CTATTTAAAAACATGATGTGCTTGAATAATCCCGTACAAATATTCAGATTTTTTATTAATTTCATCAAACACGACTAAATATACAATATAAA
ACTTTTATAGCATAAACTTGGAACGCAAAAATCCCATAATTGTGCTTAAAATCTAAATGTTAAAATATTACTAGCGGCGTCACGTCCAAGCT
CAAGTTGCTAGTAGAAGCACTAGCAGCGCGCTGACGAGTAGGGACGCTTCTAATGACCATTGATGTAACTATTTTAATATTCCAAACCAAAA
TTCATATAGTTGATGTGCTTTAATTTTTCAATTACTTTCATCGAAAACAACTAAATGTATGTTAATTTTTTTTGGCATATAAAGCCCCATAA
TCATGTTTGAAATTATGAAATGCTAGCATGTGCACTACTAGAATACAAGCTAACGTCGTCTGCGTTTACTCAAATGCTGCGAATAACAAAAT
TACATTAAATTTCTTTAAAATAGCAGAACAACCTCACATGAATCTCAAGATTGGAGATGTAACTGTGAATTGTGTACATTACATGTATACAA
ATGTTTTAAGTCATGAAAATAACAGCACTGCTATGTCTCTCTCAAACCAGACAGTTTCCCCCATCGAAACGAATCTCCCATGGATATCGATC
CCTTCGGTTTGGAAGCAACATATATCAAAACTAGCGAGTAACACACTGAAATTTTAACCACTTATTTAAGTGATCACATAACAACACTATGC
AAAGTTACAATTTTTCCAGGCACAACTTATGCTTGGCAATATAATTAAAAGAAAATAGAACTTAACTACGGCAGGAGTATATGCTTGATATA
GACTCCACTTGGTTGGAGTTAAGAAAGCACCACACATGTTGCTGGCATTGATCACCGCACCGCCTGATCAGTACATATGCCGGTGGAAATTA
ATACTACTGTTTTGCTTTTAACGATGCATGGCATGGCAGAAGGATAGATCGGAATACTACTGTTTTGCTTTTAACGATGCATGGCATGGTGG
AAGGATAGATCGGTCGTACTCAAAATTTACTCCATATCGTGTCCATGTCCGTCCCTTTTTTTGTGTTTGACAACGACGTTCTACCTACTCAC
CACTGGCCCTTCTTCCAGATCTGCACACTACACTGTGGACTGTGGTTGCTCGCACACAGGTTCCATACGTTCACACGCGAAGAAAATCACGT
CGGAATTATTTGCAGAAGCTTCTGCTCATACTAATGTAGTAGATGTGAACACTTTTTGAAAAACAATATATGTAGATGTGAACAAAGAAGCG
TCACATGCGTGCAGTGGAGTGGACGCGGCCGAGCGATCCATGATCCATCGTACGGTCCCCAGCGCCCAACCACCGGCATGCGCATGCATGCA
TGCCCCACTGGCTACGTACGACGTACGCGCATGGA**CACGTG**GATGCACGCACCACCGTCCGCCACCCTGCAATGCAATGCCGCAACACCTAT
CTTCTTCCTGGCCATCTCTCGCCCGCCGGCTATTTATACCCAGCTCCACGGAGTTCCAATCCATCTCCACAAGCGCCATCACCACACAAGCT
AATCAAGGTCTCAGCTCCTTCCGAGCTCCACGATTTCCAGCAAGCAAGCCGAAGCACTAGATCCTCG**ATG**

Short-chain dehydrogenase TIC 32 (NCBI accession no. XP_020201268.1; IWGSC accession TraesCS3D02G077500.2)

TTGCATATGGAAGCTCCACGGAGAATTGTTGTATGTGGGTTGTCAATGAGAAATGACACCTAGCTTGTGGGATCATCGGATACGGGCCCTCT
TCGATTCAAATAACTTTTCAAGGTTTTTTTTGAGAATTTTCATCCTTAAGAAAAAAAATACATGTCGGTAGTTTAAATTATATGATCATAAGG
TACAGGAGATTTTTTGTCATTTGTGCTAGATTTCAAGGAAAATTTTCATCCACTCTGGCATGTTGAGAAATCGTATGTTTCCTTATTCCCGT
GTGTTAAAATCTTGTTTAACATGAAGCGACAATGATCGCACATGACCTGTCGTGGTCCCTTCTTTTTTGTAGTACAACTACATTTGATTACC
CATTTTGCAGTACTCCTAATACGTTCGATTTGTGGCATGTTTTGATTCAAAGGAATAGTGCATGATATTTGAAGAATTTGAATCCATGGGAA
TTATTCTATGTGGATCGTTTGATTCATAGAAACGGAATTCTCATGACCTATTTGGATTGAAGGAACTTTATAATTTTCTTAGAGGATTTCAT
TGTGTAGGAACTTTTCCTATATAACCATTTGAATCATAGGATCCTGGCCACTAAATGCTTATGGAATCATTTGTACCTGGACTAATGCACAC
TTGGATCATAGGAATTGTCCCTAACTCCCTAAGGAATCATATTTACTCTATTTTGGAGAAAAGTTTCCATCCACTTAGGACCCATCTGGTTT
ACATGATTTTTGGAAACATGCAAGCATGCGTGTTTCAGTCATTAGCACAACTAGCTTCGTCATGAGGCAAGGGAGGTGAAGAACAATGCAAA
TCCTTGATATGAGAATGGTGATTGTGGGTAGTGCTTTTTCTTTGATTAATAAGATATATAACATGGGATGTGTCATGCACTGCTCCCATATT
CATTGTTGTCAGAAGATCATATCTTAGTTTGTGCTCACCTACTCCTCATTCAAAGACAGGAAGGAGAGAATATATAAGCCTTTATGCATTTA
TTTGTCACATCTCTTCTCACCTATATATAACTAGAGATGCGCGCACGATGCAAACTAACATATAGTTAGAAAGTTACAACCGACCAAGCATT
CTCATGGCTTGCATTGCAAGAGAAACCAAATACCTTCTACATAGGTTGGTCTAGAATAATTATTGGGAATGAGTGGCACCGGAAAGCAGTCT
ATTTGGATGCCCCTACATGTTAGGGCACCATCTCCAACTCATGCAATCCATGATCAATTCGCCACATAAGCAAAGCACACACACAGTAATAT
GTGTGCCACCTTATTGCCATGGAGATAGTGTGCACCATCTAGTCTTTCACTGGTTGCGGGTGGCGGCTTCCATCAAGAAAGGCCCTGGAGGC
AGTTCAACGCATACATTGTGTATCCCTCTATGTCTCGCAGCCGACATTTTCAGCAATCACTATTAGACAGAGCGCACGCAAACATGTCCTGC
ATCCTATGATTCTTCTACGCGAGAACAGTGATATCTAGTCGCAAGATGCAACTACATCAGTAGATCAACACCGTTGCACGGTAGCTGTTGAG
AGGCAAACACAGTACACTATTCTTACACCAACAAATCCTTTGATTTTTGAGAGAGCTCGATCCCTGACTTTATTTCCCGATTATTTGTAAAC
TACTCCCTCCGTTCCTAAATGTAAGTCTTTGTAGAGATTCTACTAGATGGACTACATACGGAGCAAAATGGATGAATCTATACTTAAAACGC
ATCTATATACATCCGTATGTGGTTCATAGTGGAATCTCTGCAAAGACTTATATTTAGGAACGGAGGGAGTATATGTCAACTATCTGAGAATA
GAGTCTTATTCATGTCAAAGCAAAAATGCAGCAGTTGATCAA**CACGTG**TGGACTGTGGACTCTTCTCCTCTGGATTTCAGATTCCTCCTAGG
AAAAGAAGATGCGATTTTTTGTCCAGTGCCAGAGACCACACACATGCATATTCTTCCCGTGCAAGAAGAAGGAATCCAGAGAGGAAACCTGT
TCCATCGGAATAGTAGAGTCCCGGCCCGGCCGGATTCACTCGAGAGTTTGCGTTCCGGCCGGTAGCCTGCCCGACCGCCTACTCCCCAACCA
AACTGCTGCTACCGCTCCCCGCGGCCATAAATAACCTCCTCCCCTGCGCCCGCTCCTGCACGCCTGCCTCCTCCCTCCCTCCTCCCTTCCGT
CGCGCATCACCGGAACCAATCTCGCCATCGCCTAATCCGTCCAGGTCCGTCCAGGTGAGTTCGCCAAGATTCCTCTGCTTCTTCTCCTTCTT
CTTCTTCTTGATTGGGGGCTCACATTCTGTTCCGCAGTTTCAGGAGTCGGCCCAAGAACAAG**ATG**

Type 1 non specific lipid transfer protein precursor (NCBI accession no. CAH69206.1; IWGSC accession TraesCS3B02G063500.1)

TGGAGATGCTCTAATGACGCATCCATCGGTAGTAGTAAATCATCATGAGACATGCTGCTACATTGACGGATGAGGCACTTACCATTAATAAA
AAGCCGAGTGGACTATGGCACTGAATACTTGCACAGAGGCAAATTAACATCTATTCACTCCAGGTGAACCAAGGGGACAGTGACATTTGGTG
CTGAATTACAAGTCTCCCAAGTGCACATTTTTGCTCTGTGGTAATTTAGAATGTGTATTTGTAAAACAACTGAAAATATATTAACTAAGAAT
ATATTTATATATGCATTTAGATCAAATATCGTCCGGTTTTGTACCAACTTAGTTCCATTGATATATTACTAGAGTTAACGTGGAACGATCAA

TTTCGTTTCTGAAGGTGGTGCACACATC**CACGTG**AACGAAAAAGACAGGCAAGGGTATCTTGTGAGGAAACAAGAGATAGTGTTCCTTTGTT
TTCTCTTAGAACAAGACTGGCAATGGCGTTCCCCACAAATAAATTGTAACAACAAGAACAACAACAACAATAATAATGACTATAATAGTAAC
GACGACCAAGAAGATGATGGACATAAAGGTCTTAACATGAACATGTGTCCTTTTGTGTTTTTGAGTTGCTAGAATCGTGAGATCACCTTCGG
ATAAAACATGCATGATGTACAAATAAAAATTCGAAATGTAGCTCTGAATATGGTACCAATGAATGTTAAGCTGTGATATTGGGACCAAATAA
GGCAAGGTAGATGATCCCCAGGCATGCATGACCACATGCACAACTAGATTGATATAGTTCACTTGTCGAGCTCACCATAATTAATGCACTCA
ACTACCGAAATCACTGATTAAAGAGTACTCTTTGTAAATGTCACTCACACCTTCCCATAGTGCAACATGTGTTGAACTGCATGTGCGCCACT
TGTCGCAACCTGGGATTTTTCCTTTTTCGTAGATTTTTAATTCAAAATGTTTTATCTCTCAACCATGTGTCCAAATACTGAACCATTTCACC
ATTGGATTCCTCGCGTCGAGATCTTCAAAACTAGATCGTATGTTGATAGGTTTCGACGAACTTTTTTTCACGAAAAAATCGAACGAAAAAAA
CCCGAACCGAGAGCACGGTTTTCCCTTTCCAAGAGCATGACCAAGCTTCTCGCGGAAGCAAATCCGCACCTCCACGAGAACTAAATTAGTGC
CTCTCGTGGAAGGAAAAGAACATATTTTGTCTTTTTCTGAGAGGCACGGCCGTTCTTGTCGTGGAAGCAAATCCGTGCCTCCACGAGAAGTA
AATTAGTGCCTCTCATGGTCGAAAAAATCCAAGATTTTTCCCTCTCGCATAAGCAAATTCTTGACTCCACGAGAAGTAAATCAGTACCTCTT
GTGGATGGAAAAAACATTTTTTTCCCTTTTCCAAGATGCACGGCTGTGCTTCTATTGGAAGCAAATCCGGTTTCCGCTTTGAAGCTGAGCTA
TTGGATTCGGTAGGCCAGAGCGTATTTTGGGTAAAAATGATGATGTACGTATATGCACAGTTCAATGGTCGTGCTCACCATCATAGATGCAC
TCAACTACTCCATGGTCGGCCACTATATGACTGCGTACATGTCTTAGTCTCATAGTTAATCTGCTGTGTACACGCTAATCAACCACATTCCA
CCGCTAGAACGCCTGGATGTAGTGCTTGGTCCATCCATCGGCCCAACCAAACGGTAACAACCAACCATCTCCTAAATGCATTAACACCCATA
AATCAATTCCAACATCCAACAACCTAAATTCATGCATTTGCTCGCATCAATTGAATTGGCACACGTAGACACAATACTTGACACCAAGAAAG
TACACTGTTTATTCCGAATGGAATCCCGTGATGGTAAGAAGCCCTATTTAAGCCCATGCATCCCCACGAAACTCCTCACCATTACAATCACT
TAGCAAATCTAGCTATCCCATCATCTCTGCCTGAGCTCACTACCACTACTATTGCTAGCTTGATCGAG**ATG**

Supplemental Figure S5. The promoter sequences of the genes encoding the identified proteins with known functions. These promoter sequences (about 2,000 bp) have been retrieved from IWGSC database. The ABA-responsive elements (ACGT) in each promoter are labeled with red color, and the start codon (ATG) of each gene is highlighted with black bold words. Accessions of proteins in NCBI and IWGSC databases are listed. Promoter sequences of the genes encoding dehydrin DHN 3, putative chlorophyll a-b binding protein 1C, and insulin-degrading enzyme have not been found in these two databases.
