# Supplementary material for: Functional characterization and regulatory mechanism of wheat CPK34 kinase in response to drought stress
Source: BMC Genomics. 2020 Aug 24;21:577. doi: 10.1186/s12864-020-06985-1 (PMC7444251; doi:10.1186/s12864-020-06985-1)
Supplement: Supplementary file 2 — Additional file 2: Supplemental Table S1. Meteorological parameters (rainfall, temperature, etc.) after anthesis in the experimental field. [file 12864_2020_6985_MOESM2_ESM.pdf]

Supplemental Table S1. Meteorological parameters (rainfall, temperature, etc.) after anthesis in the experimental field.

Date(year-month-day)	Sampling Timepoints	Temp. min. (°C)	Temp. max. (°C)	Weather	Rainfall (mm)
2015-04-22		27	14	Cloudy	0
2015-04-23		29	15	Clear	0
2015-04-24		28	14	Clear	0
2015-04-25		29	17	Cloudy	0
2015-04-26	5 d	29	18	Cloudy	0
2015-04-27		30	18	Cloudy	0
2015-04-28		31	16	Clear	0
2015-04-29		28	16	Clear	0
2015-04-30		29	17	Clear	0
2015-05-01	10 d	27	16	Light rain	4.2
2015-05-02		25	15	Overcast	52.6
2015-05-03		27	17	Cloudy	0
2015-05-04		24	15	Cloudy	0
2015-05-05		27	17	Cloudy	0
2015-05-06	15 d	24	14	Light rain	0
2015-05-07		26	15	Cloudy	17.1
2015-05-08		26	14	Cloudy	6.2
2015-05-09		24	13	Cloudy	0
2015-05-10		22	11	Light rain	1.1
2015-05-11	20 d	24	14	Clear	1.1
2015-05-12		29	15	Clear	0
2015-05-13		32	18	Cloudy	0
2015-05-14		27	18	Cloudy	0
2015-05-15		26	16	Clear	0
2015-05-16	25 d	28	19	Clear	0
2015-05-17		28	19	Cloudy	0
2015-05-18		32	19	Clear	0
2015-05-19		31	18	Light rain	0
2015-05-20		27	16	Clear	0
2015-05-21	30 d	24	17	Overcast	0
2015-05-22		27	17	Cloudy	0
2015-05-23		31	18	Clear	0
2015-05-24		31	18	Clear	0
2015-05-25		31	19	Clear	0
2015-05-26	35 d	28	20	Cloudy	0
2015-05-27		31	19	Clear	0
2015-05-28		31	21	Cloudy	0
2015-05-29		25	18	Light rain	0
2015-05-30		31	21	Clear	0
2015-05-31	Harvest	32	23	Cloudy	0

Notes: This table lists the meteorological parameters during the wheat grain filling period (from 22 April to 31 May, 2015) at the Experimental Farm of Agricultural Faculty of Henan Agricultural University. After from 6 to 31 d May, 2015, corresponding to 15~35 days after anthesis, there were only 25.5 mm rainfalls in this experimental site and this rainfall amounts are inadequate for the growth of wheat plants, indicating that there occurred drought stress during this period.
