# Supplementary material for: Functional characterization and regulatory mechanism of wheat CPK34 kinase in response to drought stress
Source: BMC Genomics. 2020 Aug 24;21:577. doi: 10.1186/s12864-020-06985-1 (PMC7444251; doi:10.1186/s12864-020-06985-1)
Supplement: Supplementary file 6 — Additional file 6: Supplemental Table S5. Primer sequences used in this study. [file 12864_2020_6985_MOESM6_ESM.pdf]

Supplemental Table S5. Primer sequences used in this study.

Primer name	Primer sequence (5'-3')	Usage
TaCPK34-CDS	F:CCCCTGCATTCTTGACCT	Amplifying ORF fragment
	R:ATCGCTTGCTGTTCTGTT	of *TaCPK34*
qPCR	F:GCCAAGGACCTCGTTAGAAAGA	qPCR of *TaCPK34*
	R:TCTTGAACATCTCCTTGAGCCC	
TaActin	F:AAACGAAGGATAGCATGAGGAAGC	qPCR of *TaActin*
	R:AGCGGTCGAACAACTGGTA	
TaCPK34-VIGS	F:CCTTAATTAACGCACCATCGTCGAGATCA	Construction of VIGS
	R:TATGCGGCCGCCCCGTAGTTCCGCTTCAGG	vector

Notes: *TaCPK34*-CDS, primers are used to amplify the coding sequence of the *TaCPK34* gene; qPCR, primer are used to measure the transcripts of the *TaCPK34* gene by using qPCR method; *TaCPK34*-VIGS, primers are used to amplify one fragment (219 bp) of the *TaCPK34* gene for construction of BSMV-VIGS-TaCPK34 vector.
